# A unified framework for finding differentially expressed genes from microarray experiments

**DOI:** 10.1186/1471-2105-8-347

**Published:** 2007-09-18

**Authors:** Jahangheer S Shaik, Mohammed Yeasin

**Affiliations:** 1 Department of Electrical and Computer Engineering, CVPIA Lab, University of Memphis, Memphis, TN-38152, USA; 2 Bioinformatics Program, CVPIA Lab, University of Memphis, Memphis, TN-38152, USA; 3 Biomedical Engineering, CVPIA Lab, University of Memphis, Memphis, TN-38152, USA; 4 4Center for Advanced Robotics, CVPIA Lab, University of Memphis, Memphis, TN-38152, USA; 5 Software Testing and Excellence Program University of Memphis, Memphis, TN-38152, USA

## Abstract

**Background:**

This paper presents a unified framework for finding differentially expressed genes (DEGs) from the microarray data. The proposed framework has three interrelated modules: (i) gene ranking, ii) significance analysis of genes and (iii) validation. The first module uses two gene selection algorithms, namely, a) two-way clustering and b) combined adaptive ranking to rank the genes. The second module converts the gene ranks into p-values using an R-test and fuses the two sets of p-values using the Fisher's omnibus criterion. The DEGs are selected using the FDR analysis. The third module performs three fold validations of the obtained DEGs. The robustness of the proposed unified framework in gene selection is first illustrated using false discovery rate analysis. In addition, the clustering-based validation of the DEGs is performed by employing an adaptive subspace-based clustering algorithm on the training and the test datasets. Finally, a projection-based visualization is performed to validate the DEGs obtained using the unified framework.

**Results:**

The performance of the unified framework is compared with well-known ranking algorithms such as t-statistics, Significance Analysis of Microarrays (SAM), Adaptive Ranking, Combined Adaptive Ranking and Two-way Clustering. The performance curves obtained using 50 simulated microarray datasets each following two different distributions indicate the superiority of the unified framework over the other reported algorithms. Further analyses on 3 real cancer datasets and 3 Parkinson's datasets show the similar improvement in performance. First, a 3 fold validation process is provided for the two-sample cancer datasets. In addition, the analysis on 3 sets of Parkinson's data is performed to demonstrate the scalability of the proposed method to multi-sample microarray datasets.

**Conclusion:**

This paper presents a unified framework for the robust selection of genes from the two-sample as well as multi-sample microarray experiments. Two different ranking methods used in module 1 bring diversity in the selection of genes. The conversion of ranks to p-values, the fusion of p-values and FDR analysis aid in the identification of significant genes which cannot be judged based on gene ranking alone. The 3 fold validation, namely, robustness in selection of genes using FDR analysis, clustering, and visualization demonstrate the relevance of the DEGs. Empirical analyses on 50 artificial datasets and 6 real microarray datasets illustrate the efficacy of the proposed approach. The analyses on 3 cancer datasets demonstrate the utility of the proposed approach on microarray datasets with two classes of samples. The scalability of the proposed unified approach to multi-sample (more than two sample classes) microarray datasets is addressed using three sets of Parkinson's Data. Empirical analyses show that the unified framework outperformed other gene selection methods in selecting differentially expressed genes from microarray data.

## Background

The high throughput experiments such as DNA microarrays have become one of the most popular biotechnologies to monitor the expression levels of thousands of genes simultaneously. Microarray experiments produce expression profiles measured under some experimental conditions and are normally labeled on the basis of external information such as, clinical identification of samples or expression of genes with respect to time [1]. By analyzing microarray expression profiles one can deduce information that can provide significant understanding of the mechanism of the disease under study. Sophisticated statistical techniques are required to extract relevant genes given enormous amount of microarray data. The gene selection can be a challenging issue as the microarray data is skewed with a large number of genes in one dimension and a few samples in the other dimension. There is a large volume of biological and technical noise that must be normalized to generate a more uniform measure.

The gene selection is performed typically using one of the following criteria, i) finding differential expression of genes individually (statistics based gene selection) or ii) co-expressed genes offering high discrimination between two classes of samples (clustering based gene selection). Both of these criteria lead to different computational procedures in the selection of differentially expressed genes (DEGs). A plethora of mathematical techniques have been developed for finding DEGs in microarray data, for example, [1-4]. The performances of these methods are hard to quantify and compare as they yield significantly different results on the same dataset. This problem can be attributed to the assumptions behind the methods employed for ranking as well as to the unique characteristics of the microarray data. It is widely acknowledged that no single method is adequate to produce the desired result. The fusion of the algorithms that are diverse in nature may lead to the desired result [5]. This paper proposes a gene selection method which is a blend of clustering and statistics based ranking. The gene selection is performed first by employing the two-way clustering and statistics based ranking. These ranks are converted into p-values using R-test and fused using the Fisher's omnibus criterion. The significance of the genes is analyzed next by performing false discovery rate (FDR) analysis.

The clustering-based ranking is performed using two-way clustering. The two-way clustering framework involves clustering the genes into relevant groups and then clustering the samples using the gene groups. Many different frameworks have been proposed for two-way clustering of microarray data. For example, Getz et al. [2] proposed a procedure called coupled two-way clustering by iteratively applying one way clustering within the subgroups of gene and tissue clusters from the previous iteration. Tang et al. [6] reported an inter-related clustering framework based on an iterative process that uses heuristics to define the number of clusters. McLachlan et al. [7] assume a model of distribution to cluster the genes. Theunique characteristics of microarray data limit the utility of some of these methods.

The performance of a two-way clustering framework also depends on the underlying clustering algorithm. A plethora of clustering methods have been proposed for mining microarray data [8-10]. They include but are not limited to hierarchical methods [8], self organizing maps [9], k-means clustering [10] and their variations. This paper employs an adaptive subspace iteration (ASI) based algorithm for clustering microarray data (see methods). This algorithm is well suited to handle a large number of data points. The centroids of the clusters are available as one of the outputs hence new data points may be assigned to relevant clusters with ease (dynamic data clustering). This faster computational algorithm as the results suggest complements the two-way clustering framework employed in this paper.

The performance of the statistics based algorithms on the other hand depends on the number of available samples. If the samples are less, which is true for microarray data, it is difficult to assume a distribution for the data. The ranking functions based on mean and sample variance yield inaccurate results due to the high level of noise. The statistical methods followed for finding DEGs were initially based on 2 sample t-test [11] obtained by pooling the variances from two cases [12]. The estimates used here are based on the assumption that there are a large number of samples for statistical analysis. Tusher et al. [13] pointed out that small sample variance estimates (not much variation among the samples) yield false alarms for DEGs. They introduced an additive constant to the sample variance to reduce the false detection rate. This parameter estimation was later proposed by Jeffery et al. [14] as the 90^th ^percentile of the sum of gene specific global standard errors. Mukherjee et al. [3] proposed the notion of reproducibility to minimize expected loss in determination of test statistics. The mean often is not a good representative of all the samples and may be corrupted by the outliers. Therefore, Shaik et al. incorporate Hausdorff distance into the combined adaptive ranking function to cope with the unique characteristics of the data sets and to improve the robustness of the ranking algorithm [4]. Most of these methods provide the user with only the ranks for the genes and the significance of the genes is unknown based only on the ranks.

The p-values are an indication of significance of the genes based on differential expression. This is important for feature selection studies because the p-values indicate the probability that a gene is deemed significant not by chance (FDR – false discovery rate). There are several significant studies that focus on this important issue [15-18]. However, most of the gene selection methods provide the user with only the ranking of the genes [3,7,13,14,19]. The ranks may be used to sort the genes based on differential expression from highest to lowest. The rankings do not indicate the significance of the genes. The non-availability of the p-values therefore poses problems in gene selection. The availability of p-values enables controlling the false discovery rate, which is to accept a minimum number of false positives relative to the number of rejected hypotheses. An R-test is performed in this paper to convert ranks to p-values [20].

The validation of DEGs is a challenging research issue. This paper uses 3 different methods, namely FDR analysis, clustering and visualization based methods to validate the DEGs. The ASI-based clustering algorithm [21] is employed for the clustering based validation. The steps involved in clustering-based validation can be summarized as follows:

• Find the differentially expressed genes between sample classes using the training set.

• Apply ASI algorithm to cluster the training samples using DEGs as features and verify if the clusters are consistent with different classes.

• Apply ASI algorithm to cluster the test samples with DEGs as features.

• Compare the obtained clusters with the class label information of the test classes.

• Repeat the process using all the ranking functions such as t-statistics [11], SAM, Adaptive [3], combined adaptive [4], two-way clustering [21] and the proposed unified ranking.

The application of projection based techniques by Shaik et al. [22,23] for the visualization of microarray data is found to be well suited for the multi-class microarray datasets. In this paper the 3D star coordinate projection (3DSCP) algorithm originally proposed in [22] is used for the visual validation of the DEGs. The key idea behind the application of visualization algorithms for the validation of DEGs is that if the DEGs are used as features to project the samples, the samples of different classes should be projected to distinct locations in the projected space else a random projection pattern is observed [24]. The 3DSCP algorithm is provided in additional File 1.

## Methods

This section discusses the basic formulation of the individual modules of unified framework. The overview of the unified framework is presented first.

### Unified framework

**Figure 1:**
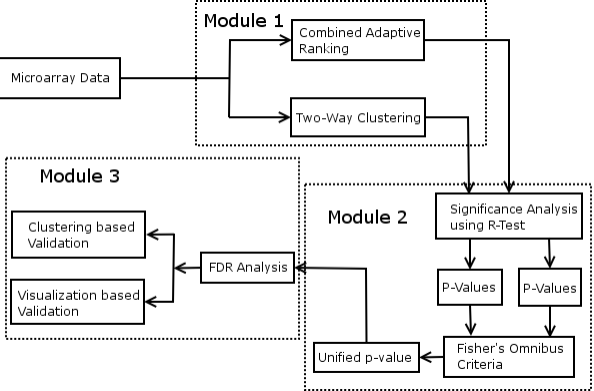
Unified Framework to find DEGs from Microarray Data.

The proposed unified framework as shown in Fig. 1 consists of three modules viz. i) Gene ranking, ii) Significance analysis of ranking and iii)Validation. The genes are first scored by employing two-way clustering framework and combined adaptive ranking. The gene with highest score is given rank 1; gene with next highest score is given rank 2 and so on for both the methods. The ranks are converted into p-values (*P*_1 _and *P*_2_) using the R-test which is discussed later. The p-values *P*_1 _and *P*_2 _are combined using Fisher's omnibus procedure to obtain the unified p-value (*U*).

U=−2∑k=1Nlog⁡Pk.     (1)
 MathType@MTEF@5@5@+=feaafiart1ev1aaatCvAUfKttLearuWrP9MDH5MBPbIqV92AaeXatLxBI9gBaebbnrfifHhDYfgasaacH8akY=wiFfYdH8Gipec8Eeeu0xXdbba9frFj0=OqFfea0dXdd9vqai=hGuQ8kuc9pgc9s8qqaq=dirpe0xb9q8qiLsFr0=vr0=vr0dc8meaabaqaciaacaGaaeqabaqabeGadaaakeaacqWGvbqvcqGH9aqpcqGHsislcqaIYaGmdaaeWbqaaiGbcYgaSjabc+gaVjabcEgaNjabdcfaqnaaBaaaleaacqWGRbWAaeqaaaqaaiabdUgaRjabg2da9iabigdaXaqaaiabd6eaobqdcqGHris5aOGaeiOla4caaa@3F35@

Here, '*N*' is the number of p-value sets (2 in this case) and '*P*_*k*_' is the set of p-values obtained using ranking procedure '*k*'. The resultant score '*U*' follows *χ*^2 ^distribution with 2N degrees of freedom. The scores are hence compared with *χ*^2 ^distribution to obtain their significance at appropriate significance level *α*. The appropriate significance level *α *is decided based on false discovery rate (FDR) analysis such that there are minimum expected percentage of false positives. The selected genes are further validated using several validation measures.

The proposed framework for selecting and validating DEGs can be succinctly summarized as follows:

• Rank the genes using the two-way clustering framework.

• Rank the genes using statistics based ranking method.

• Convert the ranks to p-values using R-test [20].

• Combine the p-values of both gene selection methods using Fisher's omnibus criterion to obtain unified score as shown in the Eq. 1.

• Select the DEGs based on FDR analysis.

• Validate the selected DEGs.

### Module 1: Gene ranking

The marker genes are generally ranked based on two criteria, i) finding differential expression of genes individually or ii) co-expressed genes offering high discrimination between two classes of tissues. Both of these criteria lead to different computational procedures in selecting DEGs as shown below:

#### Two-way clustering

**Figure 2:**
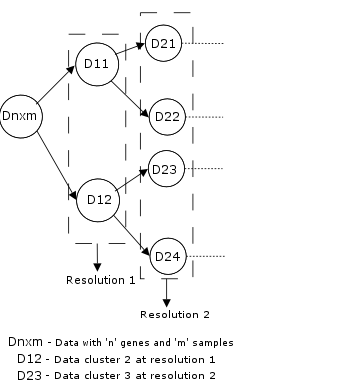
Progressive Clustering Framework to Cluster the Genes.

**Figure 3:**
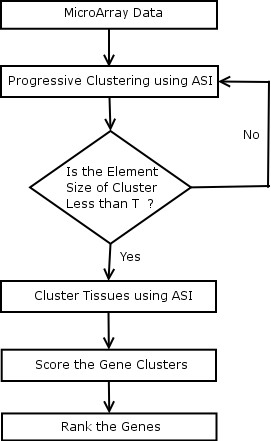
Two-Way Clustering Framework to Find DEGs from Microarray Data.

This paper employs a progressive framework as shown in Fig. 2 for clustering the genes. Unlike the traditional two-way methods which cluster the genes into specified number of clusters, the progressive framework clusters the genes into all possible resolutions. A resolution is a measure of compactness of clusters. The Higher the resolution, the compact are the clusters and vice versa. If the discriminative ability of clusters is to be studied, it must be performed at various levels of granularities. A progressive clustering algorithm provides a flexible way to achieve this goal. For example, as shown in Fig. 2, resolution 2 has more granularity than resolution 1 and so on. The clustering of the data progresses into various levels of granularity ranging from macro to micro clusters. The resolution level at which the progressive framework terminates is determined using Davies-Bouldin indices [25]. The two-way clustering framework employs the gene clusters at each resolution to cluster the samples as shown in the Fig. 3.

The sample cluster groups are compared with the sample class label information. The score for each gene cluster '*CSC*_*k*_' as shown in the Eq. 2 is determined by finding the number of correctly identified samples.

CSCkk=1M=∑i=1t∑j=1L(Ci∩Gj)     (2)
 MathType@MTEF@5@5@+=feaafiart1ev1aaatCvAUfKttLearuWrP9MDH5MBPbIqV92AaeXatLxBI9gBaebbnrfifHhDYfgasaacH8akY=wiFfYdH8Gipec8Eeeu0xXdbba9frFj0=OqFfea0dXdd9vqai=hGuQ8kuc9pgc9s8qqaq=dirpe0xb9q8qiLsFr0=vr0=vr0dc8meaabaqaciaacaGaaeqabaqabeGadaaakeaadaWfWaqaaiabdoeadjabdofatjabdoeadnaaBaaaleaacqWGRbWAaeqaaaqaaiabdUgaRjabg2da9iabigdaXaqaaiabd2eanbaakiabg2da9maaqahabaWaaabCaeaadaqadaqaaiabdoeadnaaBaaaleaacqWGPbqAaeqaaOGaeyykICSaem4raC0aaSbaaSqaaiabdQgaQbqabaaakiaawIcacaGLPaaaaSqaaiabdQgaQjabg2da9iabigdaXaqaaiabdYeambqdcqGHris5aaWcbaGaemyAaKMaeyypa0JaeGymaedabaGaemiDaqhaniabggHiLdaaaa@4D84@

Here, '*M*' is the total number of gene clusters, '*L*' is the number of different labels according to the ground truth, *C*_*i *_are the samples that are part of cluster '*i*', *G*_*j *_is the group of samples having label '*j*' according to the ground truth and (*C*_*i *_∩ *G*_*j*_) is the maximum consistency between any of the sample clusters *C*_*i *_and the samples '*G*' with labels '*j*' according to the ground truth.

#### Adaptive subspace iteration algorithm

The adaptive subspace iteration (ASI) is a subspace based method to cluster the data. It involves an optimization process that iteratively identifies the subspace structure. The following notations are used in the algorithm:

• *D*_*nxm *_is the data matrix that contains the microarray data with '*n*' genes and '*m*' samples. Also, assume that each macro cluster is divided into '*k*' number of micro clusters at each resolution level (cf. Fig. 2).

• The matrix *M*_*nxk *_is the membership matrix. Each gene has '*k*' memberships corresponding to the '*k*' clusters. The cluster to which the gene belongs has membership of 1 and rest of the memberships are 0. This enables hard clustering of the genes.

• Let *S*_*mxk *_be the subspace structure associated with each gene cluster. This subspace has adequate information about the most informative genes in the cluster. The columns of *S *determine the relevance of each sample in the formation of a cluster. Hence, (*DS*)_*nxk *_represents the projection of the data onto the subspaces.

• Let '*C*' be the projection of centroid of each gene cluster onto the subspaces given by *S*_*mxk*_. This enables calculating the distances between the genes in the subspace and to each of the centroids in the subspace to determine the relevance of each gene with each of the clusters. The relationship between the '*C*', '*S*' and '*M' *is given by,

*C *= (*M*^*T *^*M*)^-1 ^*M*^*T *^*DS*.     (3)

Here, (*M*^*T *^*M*) provides the size of the clusters (number of genes in a cluster). The diagonal elements provide the size of each cluster and off diagonal elements are zero. Hence '*C*' matrix calculates the mean of each gene cluster to estimate the centroids. These centroids are projected to subspace as shown in Eq. 3.

The objective function '*O*' is given by,

O=12‖DS−MC‖F.     (4)
 MathType@MTEF@5@5@+=feaafiart1ev1aaatCvAUfKttLearuWrP9MDH5MBPbIqV92AaeXatLxBI9gBaebbnrfifHhDYfgasaacH8akY=wiFfYdH8Gipec8Eeeu0xXdbba9frFj0=OqFfea0dXdd9vqai=hGuQ8kuc9pgc9s8qqaq=dirpe0xb9q8qiLsFr0=vr0=vr0dc8meaabaqaciaacaGaaeqabaqabeGadaaakeaacqWGpbWtcqGH9aqpdaWcaaqaaiabigdaXaqaaiabikdaYaaadaqbdaqaaiabdseaejabdofatjabgkHiTiabd2eanjabdoeadbGaayzcSlaawQa7amaaBaaaleaacqWGgbGraeqaaOGaeiOla4caaa@3B80@

Here, ||.||_*F *_is called the Frobenius norm defined as ||*A*||_*F *_= *tr*(*AA*^*T*^) where, 'tr' is the trace of a matrix. The objective function optimizes by rendering the distance between the gene cluster centroid and each of the genes in that cluster as small as possible thereby making the clusters compact. Therefore,

O=12tr[(DS−MC)(DS−MC)T]     (5)
 MathType@MTEF@5@5@+=feaafiart1ev1aaatCvAUfKttLearuWrP9MDH5MBPbIqV92AaeXatLxBI9gBaebbnrfifHhDYfgasaacH8akY=wiFfYdH8Gipec8Eeeu0xXdbba9frFj0=OqFfea0dXdd9vqai=hGuQ8kuc9pgc9s8qqaq=dirpe0xb9q8qiLsFr0=vr0=vr0dc8meaabaqaciaacaGaaeqabaqabeGadaaakeaacqWGpbWtcqGH9aqpdaWcaaqaaiabigdaXaqaaiabikdaYaaacqWG0baDcqWGYbGCdaWadaqaaiabcIcaOiabdseaejabdofatjabgkHiTiabd2eanjabdoeadjabcMcaPiabcIcaOiabdseaejabdofatjabgkHiTiabd2eanjabdoeadjabcMcaPmaaCaaaleqabaGaemivaqfaaaGccaGLBbGaayzxaaaaaa@4525@

=12[tr(DSSTDT)−2tr(DSCTMT)+tr(MCCTMT)]     (6)
 MathType@MTEF@5@5@+=feaafiart1ev1aaatCvAUfKttLearuWrP9MDH5MBPbIqV92AaeXatLxBI9gBaebbnrfifHhDYfgasaacH8akY=wiFfYdH8Gipec8Eeeu0xXdbba9frFj0=OqFfea0dXdd9vqai=hGuQ8kuc9pgc9s8qqaq=dirpe0xb9q8qiLsFr0=vr0=vr0dc8meaabaqaciaacaGaaeqabaqabeGadaaakeaacqGH9aqpdaWcaaqaaiabigdaXaqaaiabikdaYaaadaWadaqaaiabdsha0jabdkhaYnaabmaabaGaemiraqKaem4uamLaem4uam1aaWbaaSqabeaacqWGubavaaGccqWGebardaahaaWcbeqaaiabdsfaubaaaOGaayjkaiaawMcaaiabgkHiTiabikdaYiabdsha0jabdkhaYnaabmaabaGaemiraqKaem4uamLaem4qam0aaWbaaSqabeaacqWGubavaaGccqWGnbqtdaahaaWcbeqaaiabdsfaubaaaOGaayjkaiaawMcaaiabgUcaRiabdsha0jabdkhaYnaabmaabaGaemyta0Kaem4qamKaem4qam0aaWbaaSqabeaacqWGubavaaGccqWGnbqtdaahaaWcbeqaaiabdsfaubaaaOGaayjkaiaawMcaaaGaay5waiaaw2faaaaa@5752@

= *tr*(*DSS*^*T *^*D*^*T*^) - *tr*(*D*^*T *^*S*^*T *^*MC*)     (7)

Here, the first component (*DSS*^*T *^*D*^*T*^) = (*DS*)(*DS*)^*T *^gives the total deviance of the data in the subspace. The second component (*D*^*T *^*S*^*T *^*MC*) = (((*D*^*T *^*S*^*T*^)*M*)*C*) first projects the data onto the subspace as given by (*D*^*T *^*S*^*T*^). Further, the sum of distance between the centroids is estimated using (((*D*^*T *^*S*^*T*^)*M*)*C*). The objective function shown in Eq. 7 is minimized by maximizing distances between the centroids of individual clusters.

The objective function in (7) is minimized by considering first '*k*' Eigen vectors of (*D*^*T*^(*M*(*M*^*T *^*M*)^-1 ^*M*^*T *^- *I*)*D*)_1:*k *_[26]. Therefore, '*S *'is updated using Eq. 8

*S *= (*D*^*T*^(*M*(*M*^*T *^*M*)^-1 ^*M*^*T *^- *I*)*D*)_*k*_.     (8)

Please note that this feature provides dimensionality reduction and further computations are all performed in the reduced sub space. The output of the algorithm is '*M*' and '*S*'. Here, '*M*' offers the cluster memberships and '*S*'offers the weights of the samples forming the clusters defined by the matrix '*M*'. Based on the membership, the relevance of the gene with a cluster may be estimated. If the membership is 0, there is no relevance and membership 1 indicates the gene belongs to that cluster.

#### ASI algorithm

Begin clustering

Step 1: Begin Initialization

*Initialize 'M' *with zeros and randomly place 1 in each row.

Initialize 'S' with Random values such that each column adds up to 1;

End Initialization

Step 2: Project the centroids of each cluster onto the subspaces using Eq. (3);

Step 3: Compute the initial optimization value '*O*_0_' using the objective function of Eq. (7);

Step 4: Perform optimization by iteratively updating D, F, S;

Begin Optimization

While (*O*_1 _<*O*_0_)   //Continue as long as the cluster compactness increases

Step 4-1: Update '*M*' given by the formula in equation (5)

Begin Loop   //Iterate over all the features

//update the memberships by finding the relevance of a gene with each of the updated cluster centroids as shown in Eq. 9

*M*(*i*, *j*) = ((*DS*)_*i*,*j *_- *C*_:,*j*_);   j = 1...k

Min(*M*(*i*, *j*)) = 1;   j = 1...k     (9)

End loop

Step 4-3: Update '*S *'given by the formula in equation (8);

Step 4-4: Compute Step 2;

Step 4-5: Compute '*O*_1_' using equation (7);

Step 4-6: If (*O*_1 _<*O*_0_);   //Check for the terminating condition//.

*O*_0 _= *O*_1_;

End optimization

End Clustering

#### Davies-Bouldin index

Davies-Bouldin index is a measure of cluster validation metric[25]. It measures the homogeneity of the clusters by finding the ratio of the sum of intra-cluster scatter to inter-cluster scatter. The intra cluster scatter is a measure of spread of a cluster. The inter cluster scatter on the other hand is a measure of distinctiveness of the clusters. Therefore, lower the ratio of intra cluster scatter to inter cluster scatter, the better.

The intra-cluster scatter is given by

Si,q=(1|Ai|∑x∈Ai‖x−vi‖2q)1q,     (10)
 MathType@MTEF@5@5@+=feaafiart1ev1aaatCvAUfKttLearuWrP9MDH5MBPbIqV92AaeXatLxBI9gBaebbnrfifHhDYfgasaacH8akY=wiFfYdH8Gipec8Eeeu0xXdbba9frFj0=OqFfea0dXdd9vqai=hGuQ8kuc9pgc9s8qqaq=dirpe0xb9q8qiLsFr0=vr0=vr0dc8meaabaqaciaacaGaaeqabaqabeGadaaakeaacqWGtbWudaWgaaWcbaGaemyAaKMaeiilaWIaemyCaehabeaakiabg2da9maabmaabaWaaSaaaeaacqaIXaqmaeaadaabdaqaaiabdgeabnaaBaaaleaacqWGPbqAaeqaaaGccaGLhWUaayjcSdaaamaaqafabaWaauWaaeaacqWG4baEcqGHsislcqWG2bGDdaWgaaWcbaGaemyAaKgabeaaaOGaayzcSlaawQa7amaaDaaaleaacqaIYaGmaeaacqWGXbqCaaaabaGaemiEaGNaeyicI4Saemyqae0aaSbaaWqaaiabdMgaPbqabaaaleqaniabggHiLdaakiaawIcacaGLPaaadaahaaWcbeqaamaalaaabaGaeGymaedabaGaemyCaehaaaaakiabcYcaSaaa@5160@

and the inter-cluster scatter is given by,

dij,t={∑s=1p|vsi−vsj|t}1t=‖vi−vj‖t.     (11)
 MathType@MTEF@5@5@+=feaafiart1ev1aaatCvAUfKttLearuWrP9MDH5MBPbIqV92AaeXatLxBI9gBaebbnrfifHhDYfgasaacH8akY=wiFfYdH8Gipec8Eeeu0xXdbba9frFj0=OqFfea0dXdd9vqai=hGuQ8kuc9pgc9s8qqaq=dirpe0xb9q8qiLsFr0=vr0=vr0dc8meaabaqaciaacaGaaeqabaqabeGadaaakeaacqWGKbazdaWgaaWcbaGaemyAaKMaemOAaOMaeiilaWIaemiDaqhabeaakiabg2da9maacmqabaWaaabCaeaadaabdaqaaiabdAha2naaBaaaleaacqWGZbWCcqWGPbqAaeqaaOGaeyOeI0IaemODay3aaSbaaSqaaiabdohaZjabdQgaQbqabaaakiaawEa7caGLiWoaaSqaaiabdohaZjabg2da9iabigdaXaqaaiabdchaWbqdcqGHris5aOWaaWbaaSqabeaacqWG0baDaaaakiaawUhacaGL9baadaahaaWcbeqaamaalaaabaGaeGymaedabaGaemiDaqhaaaaakiabg2da9maafmaabaGaemODay3aaSbaaSqaaiabdMgaPbqabaGccqGHsislcqWG2bGDdaWgaaWcbaGaemOAaOgabeaaaOGaayzcSlaawQa7amaaBaaaleaacqWG0baDaeqaaOGaeiOla4caaa@5C8C@

Where, *v*_*i *_is the centroid of i^th ^cluster, *q*,*t *≥ 1, *q*,*t *can be selected independently of each other. For example, when t = 2, *d*_*ij*,*t *_is the Euclidean distance between '*v*_*si*_' and '*v*_*sj*_'. The |*A*_*i*_| is the number of elements in the cluster *A*_*j *_and '*x*'s are the elements of cluster *A*_*j*_.

Define Ri,qt=max⁡j∈c,j≠i{Si,q+Sj,qdij,t}.     (12)
 MathType@MTEF@5@5@+=feaafiart1ev1aaatCvAUfKttLearuWrP9MDH5MBPbIqV92AaeXatLxBI9gBaebbnrfifHhDYfgasaacH8akY=wiFfYdH8Gipec8Eeeu0xXdbba9frFj0=OqFfea0dXdd9vqai=hGuQ8kuc9pgc9s8qqaq=dirpe0xb9q8qiLsFr0=vr0=vr0dc8meaabaqaciaacaGaaeqabaqabeGadaaakeaacqqGebarcqqGLbqzcqqGMbGzcqqGPbqAcqqGUbGBcqqGLbqzcqqGGaaicqWGsbGudaWgaaWcbaGaemyAaKMaeiilaWIaemyCaeNaemiDaqhabeaakiabg2da9maaxababaGagiyBa0MaeiyyaeMaeiiEaGhaleaacqWGQbGAcqGHiiIZcqWGJbWycqGGSaalcqWGQbGAcqGHGjsUcqWGPbqAaeqaaOWaaiWabeaadaWcaaqaaiabdofatnaaBaaaleaacqWGPbqAcqGGSaalcqWGXbqCaeqaaOGaey4kaSIaem4uam1aaSbaaSqaaiabdQgaQjabcYcaSiabdghaXbqabaaakeaacqWGKbazdaWgaaWcbaGaemyAaKMaemOAaOMaeiilaWIaemiDaqhabeaaaaaakiaawUhacaGL9baacqGGUaGlaaa@5F5B@

Here, 'R' is a measure of compactness and distinctiveness of the clusters formulated as the ratio of intra cluster scatter and inter cluster scatter.

Davies-Bouldin index is defined as DB=1c∑i=1cRi,qt.     (13)
 MathType@MTEF@5@5@+=feaafiart1ev1aaatCvAUfKttLearuWrP9MDH5MBPbIqV92AaeXatLxBI9gBaebbnrfifHhDYfgasaacH8akY=wiFfYdH8Gipec8Eeeu0xXdbba9frFj0=OqFfea0dXdd9vqai=hGuQ8kuc9pgc9s8qqaq=dirpe0xb9q8qiLsFr0=vr0=vr0dc8meaabaqaciaacaGaaeqabaqabeGadaaakeaacqqGebarcqqGHbqycqqG2bGDcqqGPbqAcqqGLbqzcqqGZbWCcqqGTaqlcqqGcbGqcqqGVbWBcqqG1bqDcqqGSbaBcqqGKbazcqqGPbqAcqqGUbGBcqqGGaaicqqGPbqAcqqGUbGBcqqGKbazcqqGLbqzcqqG4baEcqqGGaaicqqGPbqAcqqGZbWCcqqGGaaicqqGKbazcqqGLbqzcqqGMbGzcqqGPbqAcqqGUbGBcqqGLbqzcqqGKbazcqqGGaaicqqGHbqycqqGZbWCcqqGGaaicqWGebarcqWGcbGqcqGH9aqpdaWcaaqaaiabigdaXaqaaiabdogaJbaadaaeWbqaaiabdkfasnaaBaaaleaacqWGPbqAcqGGSaalcqWGXbqCcqWG0baDaeqaaaqaaiabdMgaPjabg2da9iabigdaXaqaaiabdogaJbqdcqGHris5aOGaeiOla4caaa@6BE3@

Here, '*c*' is the number of clusters and '*DB*' is the measure of homogeneity of the clusters. Lower the '*DB*', more homogenous are the clusters.

#### Combined adaptive ranking

The adaptive ranking based method adopts a modification of the classical t-statistic based ranking function [24]. Let '*D*_*n*,(*m*+*k*)_' be the data matrix with '*n*' genes and '*m*' samples under one condition (say tumor class) and '*k*' samples under the other condition (say non-tumor class). The bootstrapping procedure is employed on the original dataset *D*_*n*,(*m*+*k*) _and *j *<*min(m,k) *samples are randomly selected from both cases and pooled to form the data *'D1*_*n*,2*j*_*' *('*j*' samples from each condition). The process is repeated to construct another dataset *'D2*_*n*,2*j*_*'*. The readers are encouraged to read [24] for further information on bootstrapping procedure. The ranking function of the Eq. 14 is applied independently on these two datasets to obtain the scores of the marker genes describing their differential expression. These scores are ranked and sorted from highest to lowest resulting in *R*_1 _and *R*_2 _in the Eq. 15. Since these two datasets are the subset of the original dataset '*D*_*n*,(*m*+*k*)_', they must produce similar ranking. The optimized set of parameters *θ*s which provide high consistency (Eq. 16) between the rankings are obtained using Monte Carlo simulation [24]. Since it is adequate to test the consistency using a few high ranked genes '*h*', *h *= 100 is employed in this paper. The first '*h*' high ranked genes are obtained from these two rankings resulting in two sets given by Eq. 15,

R(θ1,θ2,θ3)=d+θ1θ2σ^+θ3.     (14)
 MathType@MTEF@5@5@+=feaafiart1ev1aaatCvAUfKttLearuWrP9MDH5MBPbIqV92AaeXatLxBI9gBaebbnrfifHhDYfgasaacH8akY=wiFfYdH8Gipec8Eeeu0xXdbba9frFj0=OqFfea0dXdd9vqai=hGuQ8kuc9pgc9s8qqaq=dirpe0xb9q8qiLsFr0=vr0=vr0dc8meaabaqaciaacaGaaeqabaqabeGadaaakeaacqWGsbGucqGGOaakiiGacqWF4oqCdaWgaaWcbaGaeGymaedabeaakiabcYcaSiab=H7aXnaaBaaaleaacqaIYaGmaeqaaOGaeiilaWIae8hUde3aaSbaaSqaaiabiodaZaqabaGccqGGPaqkcqGH9aqpdaWcaaqaaiabdsgaKjabgUcaRiab=H7aXnaaBaaaleaacqaIXaqmaeqaaaGcbaGae8hUde3aaSbaaSqaaiabikdaYaqabaGccuWFdpWCgaqcaiabgUcaRiab=H7aXnaaBaaaleaacqaIZaWmaeqaaaaakiabc6caUaaa@494A@

Here,'*d*' is the difference of means for 'mean method' and Hausdorff distance between different samples for the 'Hausdorff distance method'. The σ^
 MathType@MTEF@5@5@+=feaafiart1ev1aaatCvAUfKttLearuWrP9MDH5MBPbIqV92AaeXatLxBI9gBaebbnrfifHhDYfgasaacH8akY=wiFfYdH8Gipec8Eeeu0xXdbba9frFj0=OqFfea0dXdd9vqai=hGuQ8kuc9pgc9s8qqaq=dirpe0xb9q8qiLsFr0=vr0=vr0dc8meaabaqaciaacaGaaeqabaqabeGadaaakeaaiiGacuWFdpWCgaqcaaaa@2E86@ is the square root of the sample variance.

*S*_1 _= *R*_1_(1: *h*) and *S*_2 _= *R*_2_(1: *h*).     (15)

A consistency measure is obtained by comparing these two sets

*Co *= *S*_1 _∩ *S*_2_.     (16)

The ranking *'R' *which produces highest consistency *'Co*' is considered as the best ranking. The distance measure in this ranking function was initially based on absolute difference of means [3]. Mean is not a good representative of the sample expressions and may be driven by outliers. A robust distance measure called *K*^th ^Hausdorff distance is supplemented with the mean method by Shaik et al. [4]. The rankings *R*_*M *_and *R*_*H *_are obtained for mean method and Hausdorff distance method respectively using Eq. 14 and combined to develop a fused ranking method [4] as shown in Eq. 17,

SC2=W1RM+W2RHW1+W2.     (17)
 MathType@MTEF@5@5@+=feaafiart1ev1aaatCvAUfKttLearuWrP9MDH5MBPbIqV92AaeXatLxBI9gBaebbnrfifHhDYfgasaacH8akY=wiFfYdH8Gipec8Eeeu0xXdbba9frFj0=OqFfea0dXdd9vqai=hGuQ8kuc9pgc9s8qqaq=dirpe0xb9q8qiLsFr0=vr0=vr0dc8meaabaqaciaacaGaaeqabaqabeGadaaakeaacqWGtbWucqWGdbWqdaWgaaWcbaGaeGOmaidabeaakiabg2da9maalaaabaGaem4vaC1aaSbaaSqaaiabigdaXaqabaGccqWGsbGudaWgaaWcbaGaemyta0eabeaakiabgUcaRiabdEfaxnaaBaaaleaacqaIYaGmaeqaaOGaemOuai1aaSbaaSqaaiabdIeaibqabaaakeaacqWGxbWvdaWgaaWcbaGaeGymaedabeaakiabgUcaRiabdEfaxnaaBaaaleaacqaIYaGmaeqaaaaakiabc6caUaaa@424A@

Here, *W*_1 _and *W*_2 _represent the confidence in the rankings *R*_*M *_and *R*_*H *_obtained using the consistency '*Co*' obtained from the Eq. 16.

### Module 2: Significance analysis of ranking

The ranking algorithms of the module 1 provide the user with only the relative ranks. These ranks do not indicate the significance of the rankings. Therefore, these ranks must be converted to p-values to find the significance.

#### Convert the scores to p-values

The R-test followed in [20] is followed in this paper to convert scores to p-values. This is formulated as a hypothesis testing problem. Let '*I*' be the informative genes and '*UI*' be the non-informative genes. The null hypothesis is that the gene is not informative and the alternate hypothesis is that the gene is informative. The distribution of statistics under null hypothesis is obtained as follows (Please see [20] for more details):

• Obtain the ranks of the genes using the scores for 'I' iterations using bootstrapping. The value I = 25 is often adequate as indicated in [20].

• Construct the distribution of statistics under null hypothesis from consistently high ranked (insignificant) genes.

• The median rank (*r*) of each gene is obtained (in [20] mean rank was computed).

• The p-value of each gene is obtained by finding *p*(*r*_*i*_/*g *∈ *UI*) i.e. the probability of the ranking of gene '*r*_*i*_' given the gene belongs to null-hypothesis. The null hypothesis is that gene is uninformative therefore lower the probability under null hypothesis, more significant is the gene.

#### False discovery rate (FDR) analysis

The FDR analysis is the process of selecting the DEGs such that there are minimum possible expected false positives. Let '*S*_*g*_' be the number of selected DEGs at significance level *α *and let *V *be the number of false positives among the selected DEGs. The FDR as proposed by Storey and Tibshirani [17] is given by,

FDR=E(VSg|Sg>0)p(Sg>0).     (18)
 MathType@MTEF@5@5@+=feaafiart1ev1aaatCvAUfKttLearuWrP9MDH5MBPbIqV92AaeXatLxBI9gBaebbnrfifHhDYfgasaacH8akY=wiFfYdH8Gipec8Eeeu0xXdbba9frFj0=OqFfea0dXdd9vqai=hGuQ8kuc9pgc9s8qqaq=dirpe0xb9q8qiLsFr0=vr0=vr0dc8meaabaqaciaacaGaaeqabaqabeGadaaakeaacqWGgbGrcqWGebarcqWGsbGucqGH9aqpcqWGfbqrdaqadaqaamaalaaabaGaemOvayfabaGaem4uam1aaSbaaSqaaiabdEgaNbqabaaaaOGaeiiFaWNaem4uam1aaSbaaSqaaiabdEgaNbqabaGccqGH+aGpcqaIWaamaiaawIcacaGLPaaacqWGWbaCcqGGOaakcqWGtbWudaWgaaWcbaGaem4zaCgabeaakiabg6da+iabicdaWiabcMcaPiabc6caUaaa@4685@

The FDR provides the expected proportion of false positives among the selected DEGs where the number of selected genes is greater than 0. In this paper *α *is selected such that FDR is minimized. If the ground truth information about the DEGs is available, the performance of ranking algorithms may be compared using the performance analysis curves.

### Performance analysis curves

The performance analysis curves are employed to study the performance of different ranking algorithms. The problem at hand is a binary classifier where the gene is either differentially expressed or not differentially expressed. There are four possible alternatives that may be obtained from the classifier viz. true positives (TPs), false positives (FPs), true negatives (TNs) and false negatives (FNs). The TPs are the number of true DEGs among the selected DEGs *S*_*g*_. The FPs are the number of true non-DEGs among *S*_*g*_. Alternatively, TNs are the total number of true non-DEGs among the genes deemed insignificant by the algorithm where as the FNs are the total number of true DEGs among the genes deemed insignificant. If the labels for the genes (differentially expressed/ non-differentially expressed) are available, which is true for artificial microarray datasets employed in this study, it is possible to accurately find TPs, FPs, TNs and FNs. The plot of TPF vs FPF hence, enables the comparison of performance of various classifiers employed in the study. Each of the 50 artificial datasets employed in this paper is used as an instance for building the performance curves. The TP, FP, TN and FN are added at each instance for 50 artificial datasets. The true positive fraction (TPF) is obtained by using the formula TPF = TP/(TP+FN) and false positive fraction (FPF) by using the formula FPF = FP/(FP+TN). These TPFs and FPFs are plotted to build the performance analysis curves.

### Artificial microarray datasets

Two different models are employed to generate artificial microarray datasets viz. i) Lognormal model [27] and ii) Asymmetric Laplace distribution [28]. Each artificial dataset is created to have 2050 genes with 10 samples under each of the two conditions. The first 50 genes are rendered differentially expressed and the rest 2000 are rendered non-DEGs. This process enables class labels for genes (DEGs or non-DEGs) for each generated artificially generated microarray dataset which can be used as ground truth to quantitatively assess the performance of different algorithms used in this study.

#### Lognormal model

A lognormal distribution-based model is used to generate artificial microarray datasets as proposed in [27]. The artificial microarray datasets are generated based on a multivariate lognormal model. The means under both conditions for the DEGs, are set to a fixed value and for the non-DEGs, means under one condition are set to zero and for other condition are drawn from *N (3, 1)*. Unequal variances following a Gamma distribution are used for DEGs as reported in the literature [3,27]. The parameters used for generating the artificial microarray datasets are shown in table 1.

**Table 1:** Parameters for Generating Artificial Microarray Datasets

Tissue Type		Normal tissues (condition1)	Abnormal tissues (condition 2)
Non- DEGs	mean	0	0
	variance	Gamma distribution with mean 2, variance 2	
DEGs	mean	0	Normal distribution mean 3, variance 1
	variance	Gamma distribution with mean 3, variance 2	Gamma distribution with mean 2, variance 2

DEGs, Differentially Expressed Genes; Non-DEGs, Non-Differentially Expressed Genes.

#### Asymmetric laplace distribution model

The artificial microarray datasets are created using the same procedure employed for lognormal distribution but by using an Asymmetric Laplace distribution as reported in [28]. The mean and variance of the DEGs and Non-DEGs are approximated using the parameters in table 1. The sample size was set to 12.

## Results

The performance of the proposed unified framework for finding DEGs from microarray datasets is evaluated using two models of simulated microarray datasets (50 artificially generated microarray datasets each [3,4,27]) as well as six real cancer and Parkinson's microarray datasets [29-31]. Artificial datasets with ground truth information are used for the comparison of performance of unified framework with other gene selection methods. The performance of various gene selection algorithms [3,11,13] is further compared with the proposed method using real microarray datasets in the selection of DEGs.

### Artificial microarray datasets

**Figure 4:**
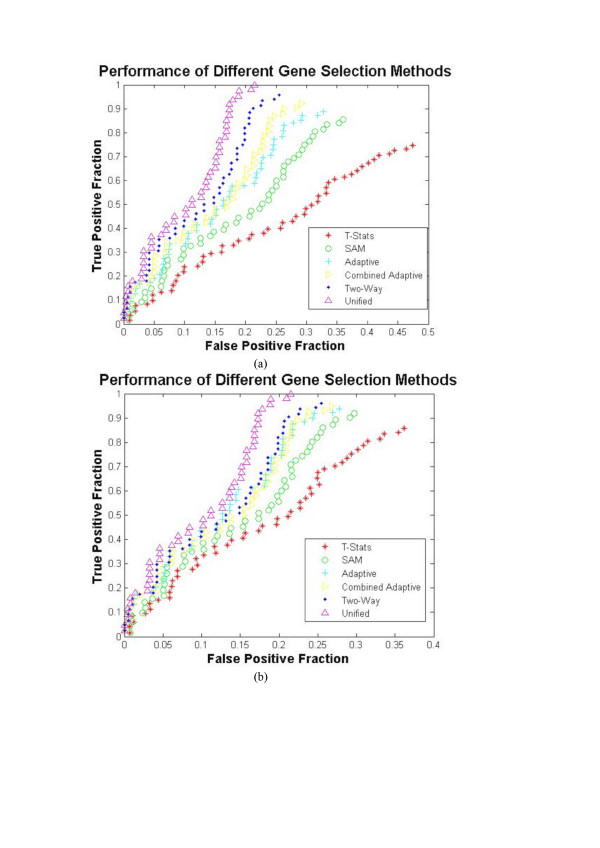
The Performance curves for various Gene Selection Methods using Artificial Microarray Datasets.

A lognormal distribution and asymmetric Laplace distribution model are used to generate artificial microarray datasets as proposed in [27,28]. The steps involved in the analysis of artificial microarray datasets can be succinctly summarized as follows:

1. Generate artificial microarray dataset such that the first 50 genes are rendered differentially expressed and next 2000 are non-differentially expressed (see methods).

2. Find the ranks using module 1 of the unified framework and convert them to p-values using the R-test.

3. Merge the p-values and obtain the unified p-value using Fisher's omnibus criteria.

4. Perform FDR Analysis.

5. Compare the DEGs with the ground truth to obtain TPF and FPF (see methods).

6. Repeat the steps 1–5 for all 50 artificial microarray datasets to obtain performance curves as shown in Fig. 4.

The unified framework, its individual modules and other well known techniques such as the t-statistics [24,32], significance analysis of microarrays [13], adaptive ranking [3], combined Adaptive ranking [4] and two-way clustering using ASI [21] are employed to find DEGs of 50 artificially generated microarray datasets. The R-test [20] is employed to convert ranks to p-values for the gene selection methods that do not offer p-values. The Fig. 4 shows the performance curves (see methods) of various well known methods and the proposed unified approach using lognormal model and asymmetric Laplace model. Analyzing the values in the performance plots, it can be inferred that the proposed unified approach outperforms the other gene selection methods in finding the DEGs from the artificial microarray data.

### Leukemia microarray dataset

Gene expressions of approximately 6817 genes are used to classify two types of acute Leukemia viz. Acute Lymphoid Leukemia (ALL) and Acute Myeloid Leukemia (AML). The data consists of 47 (38 B-cell and 9 T-cell) cases of ALL and 25 cases of AML samples. The data is divided into a training class containing 38 samples (27 ALL and 11 AML) and a test class containing 34 samples of tissues (20 ALL and 14 AML). The class labels for the training and test samples are available from Golub et al. [30]. The pre-processing steps proposed by Golub et al. resulted in 3571 genes, the rest of the genes are considered noise and therefore eliminated.

#### Gene selection and statistical validation

The various well known gene selection methods are applied on the training set and the p-values of the genes are obtained. For the gene selection methods which offer only ranking, the R-test is employed to obtain the p-values. The Table 2 shows the FDR analysis for the leukemia training dataset. As shown in Table 2, the unified framework recorded less percentage of false positives at various levels of *α *(6.8%–16.92%). This indicates the improved performance of the unified framework over other gene selection methods. The top 300 low ranked genes obtained using the unified framework is provided as a supplementary document (See additional file 2). The first 52 genes are selected at significance level 0.001 as shown in the Table 2 because it offered minimum expected percentage of false positives (6.8%).

**Table 2:** FDR Analysis of Leukemia Dataset

AlphaValue	t-statistics		SAM		Adaptive		Combined Adaptive		Two-Way		Unified	
	GS	%FP	GS	%FP	GS	%FP	GS	%FP	GS	%FP	GS	%FP

0.01	154	23.19	171	20.88	183	19.51	189	18.89	191	18.7	211	16.92
0.005	94	18.99	103	17.33	119	15	121	14.76	117	15.26	147	12.15
0.001	29	12.31	34	10.5	41	8.71	41	8.71	43	8.3	52	6.8

GS, Genes Selected; FP, Percentage of False Positives.

#### Comparison of the obtained DEGs with DEGs obtained by Golub et al. [30]

The 52 significantly expressed genes obtained using the unified framework are compared to the DEGs obtained by Golub et al. [30] (see additional file 3). There are 24 genes common to the genes found by Golub et al. This shows that the genes obtained by the unified framework are not significantly different from those obtained by Golub et al. It also shows that there are many genes selected by the unified framework that were not considered significant by Golub et al. It has already been statistically validated that unified framework offered less percentage of expected false positives and hence the genes selected using unified framework are considered to be relevant.

#### Clustering-based validation

The ASI-based clustering algorithm [21] and the steps outlined in the background section are employed for validation using clustering. The training samples are clustered using the DEGs obtained for individual methods. The obtained sample clusters are compared with the class labels of the samples. The Table 3, row 2 shows the number of correctly identified samples. As shown in the Table 3, the DEGs obtained by the unified framework offered 100% accuracy in the identification of training sample classes. The two-way framework also offered 100% accuracy in identification of the labels of training samples. Additional validations are performed to assess the performance of the individual methods.

**Table 3:** ASI Classification of Leukemia Samples using the DEGs

Gene Selection Method	Samples	t-statistics	SAM	Adaptive Ranking	Combined Adaptive Ranking	Two-way Clustering	Unified Ranking
Training	38	33	35	36	36	38	38
Testing	34	25	28	29	30	30	33

Samples, The total number of samples for training and testing; Other Columns, Number of samples classified correctly by individual methods; Example, number of training samples classified correctly by using t-statistics is 33 out of 38; The same procedure is followed for other columns of table 3 and for the tables 5, 7, 9 and 11.

The ASI algorithm is further applied to cluster the test samples using the DEGs obtained through respective methods. It is evident from the row 3 of Table 3 that the DEGs obtained using the unified framework classified the AML and ALL samples better (97.06%) than the DEGs obtained using the other methods. This also shows the improved performance of unified framework over the other methods as shown in Table 3.

#### Visualization-based validation

**Figure 5:**
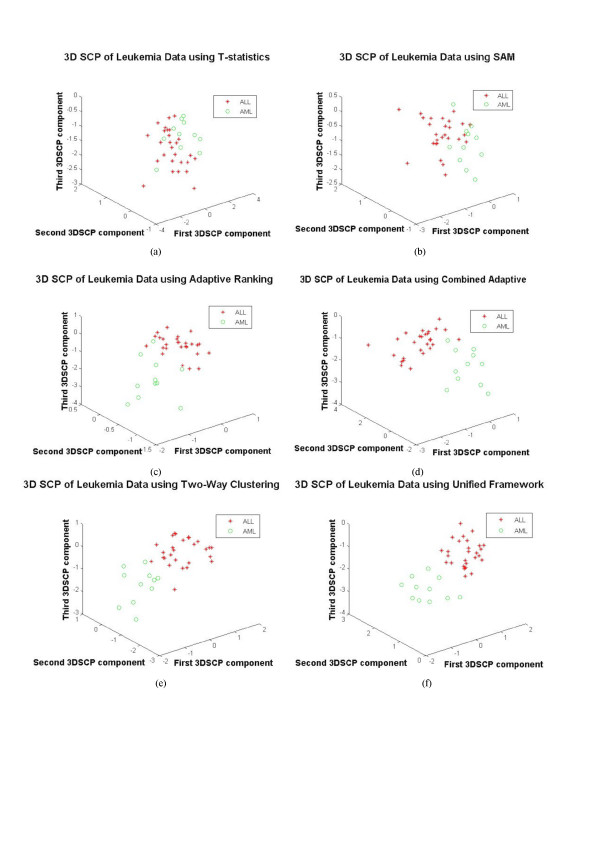
**The 3DSCP Projection of ALL and AML Samples using DEGs as Features using Various Gene Selection Methods for Leukemia Dataset**. a) t-statistics, b) SAM, c) Adaptive Ranking, d) Combined Adaptive Ranking, e) Two-Way Clustering and f) Unified Framework

The idea behind visualization-based cross validation is that if the genes obtained using a gene selection method are differentially expressed, they should separate the sample cases of different classes in the projected space [24]. The Fig. 5 shows the visualization of samples using DEGs as features using a 3D star coordinate projection algorithm (3DSCP). Comparing the Figs. 5(a) to 5(f) it may be seen that the unified framework offered clear differentiation between different sample cases. Although the two-way clustering and unified approach identified all the samples correctly as shown in the Table 3 for training samples, comparing the Figs. 5(e) and 5(f), it may be seen that the unified framework offered much clear separation between the samples of different cases. This evidence suggests that better gene selection is achieved using the unified framework.

### Gastric cancer microarray dataset

The objective of this study is to identify genes distinguishing primary gastric cancers and metastatic gastric cancers from neoplastic gastric cancers which are otherwise morphologically indistinguishable. Approximately 30300 genes are used to study expression patterns of 90 primary gastric cancers and 22 neoplastic gastric cancers. The preprocessing steps indicated by Chen et al. [29] are employed resulting in 5200 genes for further study.

The two bootstrapped datasets are created from the original dataset, one for training and one for testing. The training data has randomly selected 60 primary samples and 12 neoplastic samples where as the test data has randomly selected 30 primary samples and 10 neoplastic samples. The experimental design used for the Leukemia dataset is followed for the analysis of the Gastric cancer dataset.

#### Gene selection and statistical validation

The gene selection is performed such that there is minimum percentage of expected false positives. As shown in the Table 4, the unified framework recorded less percentage of false positives (2.48%-11.98%) than the other gene selection methods at various levels of *α*. The full list of better ranked genes can be accessed from additional file 4.

**Table 4:** FDR Analysis of Gastric Cancer Dataset

AlphaValue	t-statistics		SAM		Adaptive		Combined Adaptive		Two-Way		Unified	
	GS	%FP	GS	%FP	GS	%FP	GS	%FP	GS	%FP	GS	%FP

0.01	417	12.47	398	13.07	397	13.10	406	12.81	414	12.56	434	11.98
0.005	299	8.70	283	9.19	279	9.32	288	9.03	294	8.84	325	8
0.001	173	3.01	189	2.75	166	3.13	175	2.97	187	2.78	210	2.48

GS, Genes Selected; FP, Percentage of False Positives.

#### Comparison of the DEGs to the significant genes obtained by Chen et al. [29]

The DEGs found using the unified framework are compared against the DEGs found by Chen et al. [29]. The 204 genes out of 210 genes found by unified algorithm are common to the DEGs found by Chen et al. [29]. The list of common genes may be accessed through additional file 5. It may be seen that most of the genes found using the unified framework were present in the list of 3000 genes found significant by Chen et al. The improved performance of the unified framework may be attributed to the rejection of most of the genes deemed significant by Chen et al. This is one of the advantages of FDR analysis which focuses not only on the selection of DEGs but also on the rejection of the insignificant genes.

#### Clustering-based validation

The method similar to clustering based validation for leukemia dataset is followed for gastric cancer dataset. As shown in Table 5, row 2, the DEGs obtained by the unified framework offered 100% accuracy in the identification of the training samples which was not achieved by DEGs obtained by other methods. It may also be seen from the row 3 of the Table 5, that DEGs obtained using the unified framework identified the primary and neoplastic samples from the test set better than the DEGs obtained using the other gene selection methods (97.5%).

**Table 5:** ASI Classification of Gastric Cancer Samples using the DEGs

Gene Selection Method	Samples	t-statistics	SAM	Adaptive Ranking	Combined Adaptive Ranking	Two-way Clustering	Unified Ranking
Training	72	64	67	67	69	69	72
Testing	40	28	34	33	35	33	39

#### Visualization-based validation

**Figure 6:**
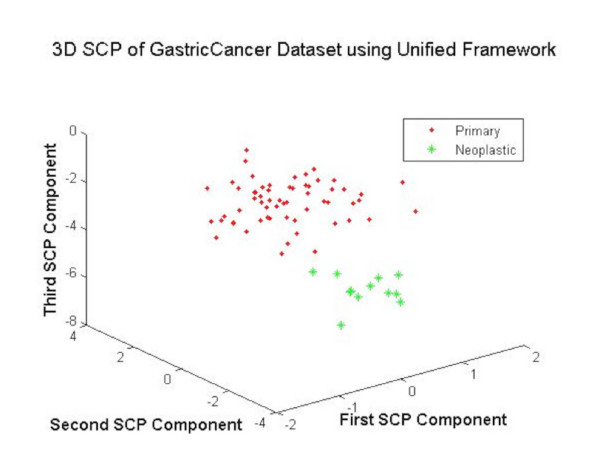
The 3DSCP Projection of Primary and Neoplastic Samples using DEGs as Features using Unified Framework.

The training samples from the Gastric cancer dataset are projected using DEGs as features for visual validation of the DEGs. The Fig. 6 shows that the unified framework offered much clear separation between the samples of different cases with no overlap between the elements of two classes. This evidence suggests the better gene selection obtained using the unified framework.

### Colon cancer microarray dataset

The Affymetrix oligonucleotide array complementary to more than 6500 human genes are used in this study. The gene expression is studied using 40 tumor samples and 22 normal samples. The preprocessing of this dataset resulted in 2000 interesting genes which have been used as input to the gene selection algorithms.

The analysis is performed by first dividing the data into training and test sets. The training data has 25 tumor samples and 12 normal samples selected randomly where as the test data has 15 tumor samples and 10 normal samples selected randomly. The steps similar to experimental design followed for Leukemia dataset is used for the analysis of this dataset.

#### Gene selection and statistical validation

The Table 6 shows the percentage of expected false positives for various gene selection methods for different values of *α*. The unified framework as shown in the Table 6 offered improved performance in the selection of DEGs. The full list of better ranked genes can be accessed from additional file 6. These genes are obtained for *α *= 0.001 which offered minimum expected percentage of false positives (3.03%).

**Table 6:** FDR Analysis of Colon Cancer Dataset

AlphaValue	t-statistics		SAM		Adaptive		Combined Adaptive		Two-Way		Unified	
	GS	%FP	GS	%FP	GS	%FP	GS	%FP	GS	%FP	GS	%FP

0.01	211	9.48	233	8.58	221	9.05	218	9.17	211	9.48	236	8.47
0.005	121	8.2	119	8.4	113	8.85	117	8.55	124	8.06	134	7.46
0.001	48	4.17	54	3.7	38	5.26	42	4.76	57	3.51	66	3.03

GS, Genes Selected; FP, Percentage of False Positives.

#### Comparison of the DEGs with earlier works

A list of significantly differentially expressed genes is not available from Alon et. al for comparison. However, the comprehensive analysis on this dataset is performed by Su et al. [33]. The procedure involves ranking the genes using 8 different measures viz. t-test, information gain, sum of variances, twoing rule, gini index, sum minority, max minority and ID SVM. The rankings are then fused to obtain a list of 100 better ranked genes [33]. This list of 100 ranked genes is compared with the list of 66 genes obtained using the unified framework. The 51 genes out of 66 genes were among the top 100 genes obtained using the 'rankgene' method. The rank gene method did not employ any FDR analysis for gene selection, it merely lists the top 100 genes.

#### Clustering-based validation

The Table 7, row 2 shows the number of samples identified correctly by the gene selection methods. As shown in the Table 7, the unified framework performed relatively better in the identification of training samples. The performance of the gene selection methods is also compared by using the test set. The Table 7, row 3 shows that DEGs obtained using the Unified framework performed better than DEGs obtained using other gene selection methods.

**Table 7:** ASI Classification of Colon Cancer Samples using the DEGs

Gene Selection Method	Samples	t-statistics	SAM	Adaptive Ranking	Combined Adaptive Ranking	Two-way Clustering	Unified Ranking
Training	37	31	33	31	33	32	35
Testing	25	19	22	22	21	21	24

#### Visualization-based validation

**Figure 7:**
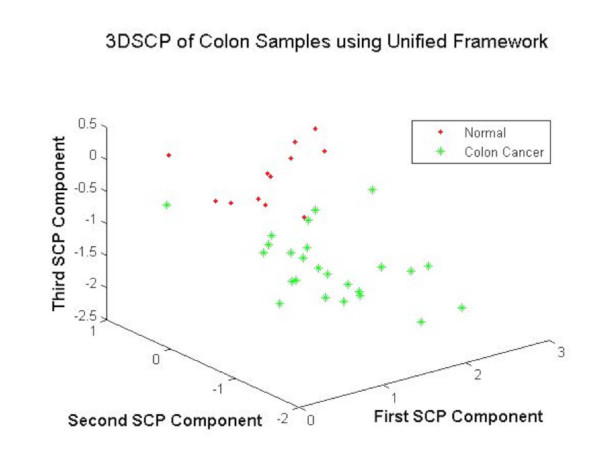
The 3DSCP Projection of Normal and Tumor Samples using DEGs as Features using Unified Framework.

The training samples from the colon cancer dataset are projected using DEGs as features for visual validation of the DEGs. The Fig. 7 shows that the unified framework performed better in the separation of colon cancer samples using the DEGs as features. The validation using 3DSCP shows the better performance of unified framework in the robust selection of DEGs.

### Parkinson's dataset

The Parkinson's dataset is employed to extend the application of two sample gene selection methods to multi-sample experiments. Three sets of microarray data are available for this model from Miller et al. [31]. The first dataset is obtained using Codelink Mouse uniSet I bioarrays. The other two are obtained using Affymetrix array data analyzed using Affymetrix Microarray Suite software(MAS 5) and Model Based Expression Index (MBEI) using dChip software. The data consists of three treatment groups MC (saline treated mouse control), MME (mouse MPTP early) and MML (mouse MPTP late). Each group has four set of samples obtained at different times after MPTP administration using 12588 genes. The performance of different gene selection methods is evaluated for the comparison of MC and MME, MC and MML groups for all the three datasets. This pattern of comparison provides DEGs at different times. This also provides information about the DEGs at the early stage that stayed differentially expressed at late stage.

#### Codelink mouse uniSet I bioarrays

##### Experimental design

• Find the p-values for the genes based on differential expression between the MC and MME groups and MC and MML groups.

• Merge the two sets of p-values using Fisher's Omnibus criterion [34].

• Perform FDR analysis and select the genes with significant differential expression such that there is minimum percentage of expected false positives.

• Apply the ASI algorithm to cross validate the DEGs.

• Repeat the process for each gene selection method.

##### Gene selection and statistical validation

The thresholding process by Golub et al. [30] on the codelink mouse uniset1 bioarrays resulted in 2347 genes for further analysis. The gene selection methods are employed for finding the p-values for the genes based on differential expression between MC and MME groups and MC and MML groups. The FDR analysis is performed on the merged p-values. The Table 8 shows the FDR analysis of different gene selection methods. Table 8 reveals that the unified framework shows improved performance in selecting the DEGs from the Parkinson's Dataset (13.8% false positives). The DEGs obtained using the unified framework at *α *= 0.001 are provided in the additional file 7.

**Table 8:** FDR Analysis of Parkinson's Dataset using CodeLink BioArrays

AlphaValue	t-statistics		SAM		Adaptive		Combined Adaptive		Two-Way		Unified	
	GS	%FP	GS	%FP	GS	%FP	GS	%FP	GS	%FP	GS	%FP

0.01	51	46.07	52	45.13	57	41.18	57	41.18	51	46.07	56	41.91
0.005	25	46.9	29	40.4	28	41.8	28	41.8	29	40.4	31	37.8
0.001	14	16.76	15	15.65	14	16.76	16	14.67	15	15.65	17	13.81

GS, Genes Selected; FP, Percentage of False Positives.

##### Clustering-based validation of codelink data

The Table 9, row 2 shows the number of samples identified correctly by the gene selection methods. As shown in the Table 9, the unified framework identified all the samples correctly.

**Table 9:** Cross Validation of Parkinson's Datasets using Training Samples

Gene Selection Method	Samples	t-statistics	SAM	Adaptive Ranking	Combined Adaptive Ranking	Two-way Clustering	Unified Ranking
Codelink	12	10	10	9	11	10	12
MAS05	12	10	10	10	10	11	12
Dchip	12	9	9	9	10	11	12

##### Visualization-based validation

**Figure 8:**
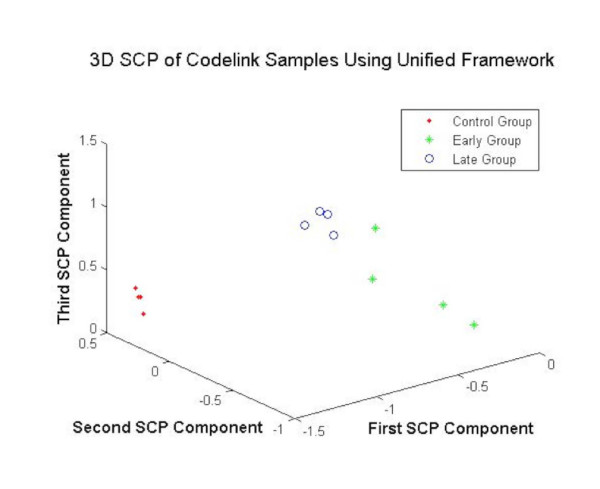
The 3DSCP Projection of MC, MME and MML Samples using DEGs as Features using Unified Framework for CodeLink Parkinson's Dataset.

The DEGs obtained using the various gene selection methods are projected by employing the 3DSCP algorithm. The Fig. 8 shows the projection of MC, MME and MML samples using the DEGs obtained by using different gene selection methods as features. As shown in the Fig. 8, the DEGs obtained from unified framework yield clear separation between different sample cases showing the validity of the selected genes.

#### Affymetrix using MAS 05

##### Gene selection and statistical validation

The irrelevant genes are first filtered out by employing the filtering process by Golub et al. [30] resulting in 2433 genes for further analysis. The experimental design used for the codelink data is followed for the MAS 05 data. The FDR analysis of different gene selection methods as shown in the Table 10 shows improved performance in selection of DEGs from the Parkinson's MAS 5 Dataset (16.71% false positives). The DEGs obtained using the unified framework at *α *= 0.001 are provided in the additional file 8.

**Table 10:** FDR Analysis of Parkinson's Dataset using Affymetrix MAS05

AlphaValue	t-statistics		SAM		Adaptive		Combined Adaptive		Two-Way		Unified	
	GS	%FP	GS	%FP	GS	%FP	GS	%FP	GS	%FP	GS	%FP

0.01	46	50.87	46	50.87	48	48.75	48	48.75	49	47.76	51	45.88
0.005	25	46.8	27	43.34	25	46.8	28	41.8	28	41.8	31	37.75
0.001	12	19.5	13	18	11	21.27	12	19.5	14	16.71	14	16.71

GS, Genes Selected; FP, Percentage of False Positives.

##### Clustering-based validation of MAS 05 data

The Table 9 shows the number of samples identified correctly by the gene selection methods. As shown in the Table 9, row 3, the unified framework performed relatively better in the validation of the samples (100% accuracy).

##### Visualization-based validation

**Figure 9:**
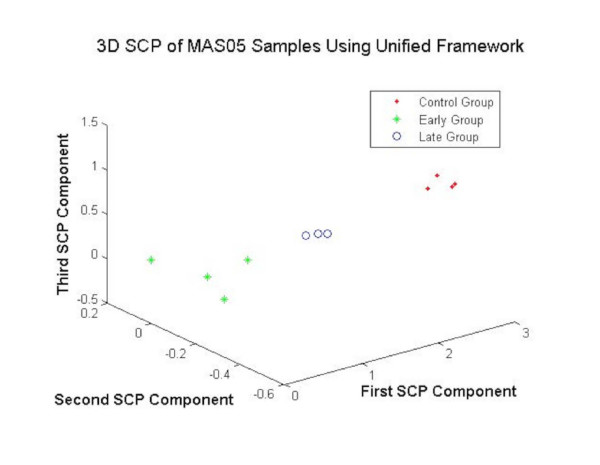
The 3DSCP Projection of MC, MME and MML Samples using DEGs as Features using Unified Framework for MAS 05 Parkinson's Dataset.

The Fig. 9 shows the projection of MC, MME and MML samples using the DEGs obtained by using different gene selection methods as features for the MAS05 data. The unified framework offered clear separation between the data points in the projected space as shown in Fig. 9 showing the validity of the proposed approach.

#### Affymetrix using dchip

##### Gene selection and statistical validation

The DChip data is first filtered to remove irrelevant genes using the method by Golub et al. [30] resulting in 2179 genes for further analysis. The experimental design employed for codelink data is followed for the dChip data. The Table 11 as shown in Table 11 offered improved performance in selection of DEGs than most of the gene selection methods (12.8% false positives). The DEGs obtained using the unified framework at *α *= 0.001 are provided in additional file 9.

**Table 11:** FDR Analysis of Parkinson's Dataset using Affymetrix dChip

AlphaValue	t-statistics		SAM		Adaptive		Combined Adaptive		Two-Way		Unified	
	GS	%FP	GS	%FP	GS	%FP	GS	%FP	GS	%FP	GS	%FP

0.01	49	44.47	50	43.58	52	41.9	52	41.9	49	44.47	54	40.35
0.005	33	33	35	31.1	35	31.1	35	31.1	33	33	37	29.43
0.001	15	14.53	15	14.53	15	14.53	16	13.62	14	15.56	15	13.22

GS, Genes Selected; FP, Percentage of False Positives.

##### Clustering-based validation of dchip data

The samples are clustered by employing the DEGs obtained using various gene selection methods (for *α *= 0.001). The obtained sample clusters are compared with the class labels of the samples (MC, MME and MML). The Table 9 shows that all samples (100%) are correctly identified using the proposed unified framework.

##### Visualization-based validation

**Figure 10:**
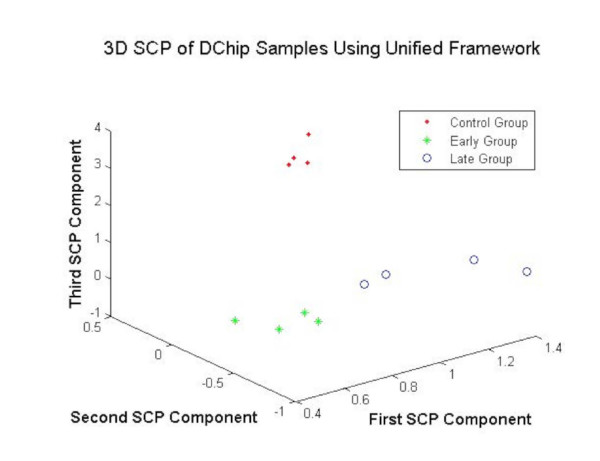
The 3DSCP Projection of MC, MME and MML Samples using DEGs as Features using Unified Framework for dChip Parkinson's Dataset.

The Fig. 10 shows the projection of MC, MME and MML samples using the DEGs at *α *= 0.001. The Analysis reveals that the DEGs obtained using the unified framework yielded good separation between the samples of various classes as shown in the Fig. 10. This evidence suggests better gene selection using the proposed method.

## Discussion

This paper presents a unified framework of gene selection and their validation. The fusion of two different gene selection algorithms viz. two-way clustering and combined adaptive ranking is performed to rank the genes. The two-way framework finds the differential expression of the co-expressed genes. The progressive framework using ASI algorithm is employed to cluster the gene dimension. This presents the gene clusters at different resolutions which may be analyzed for differential expression. The clusters at different resolutions may be tested for differential expression. The number of resolutions for progressive framework is determined using the Davies-Bouldin Index.

Most of the ranking functions employed in this study for gene selection provide the user with only the relative ranking of the genes. These ranks enable sorting the genes based on differential expression but they do not indicate the significance of genes. The R-test presents a means of converting ranks into a measure of significance (p-values). The gene rankings using module1 are converted into p-values using R-test and fused using Fisher's omnibus criterion. The FDR analysis is further applied on the fused p-values. The FDR analysis enables judicious selection of DEGs by providing a balance between the genes selected and expected percentage of false positives. For example, at *α *= 0.001 the percentage of false positives for gastric cancer dataset using the unified framework is 2.48%. This indicates that out of 210 genes there is a possibility of only 5 genes (210*2.4% = 5) to have occurred by chance.

The real datasets are divided into two categories i) Two sample experiments with a large number of samples and ii) Multi sample experiments with small number of samples. For the first category, emphasis is made on the validation techniques. The data is divided into training and testing sets. The DEGs are obtained by employing the training set and three fold validations are performed. The improvement in statistical power for the selection of DEGs is first shown with the aid of FDR analysis. The clustering based cross validation of the DEGs is performed next by clustering the training and test samples and evaluating the performance. Finally, a visualization based cross validation is performed to show the separability of samples in the projected space. The aim of the second category of the real datasets is to show the extensibility of the proposed approach to multi-sample experiments. Due to the non-availability of large number of samples, the validation is performed on only the training set by employing the clustering and visualization based algorithms. The clustering-based validation approaches clearly showed the better performance of unified framework over the other algorithms. Further, the visualization based validation demonstrated that the DEGs obtained using the unified framework offered much clear separation between the samples of the different classes than the DEGs obtained using the other methods.

## Conclusion

A unified framework for finding DEGs from microarray data is developed and empirically evaluated. The judicious combination of the three different modules is used to build the unified framework. The performance of the unified framework is compared with other well known gene selection algorithms. The performance analysis curves using 50 artificial microarray datasets each following two different distributions indicate the superiority of the unified framework over the other reported algorithms. Further analyses on 6 real cancer datasets show the similar improvement in performance. The comprehensive validation of the DEGs is presented using the first three real datasets. The robustness in the selection of genes is first presented using FDR analysis for various methods used in the study. The clustering based validation is presented next by analyzing the clustering of training and test samples using ASI algorithm. Finally, a visualization based validation is performed. The scalability of the proposed unified approach to multi-sample experiments is demonstrated using the Parkinson's datasets. Empirical analyses on artificial and real microarray datasets illustrate the efficacy of the proposed unified framework in finding the DEGs.

## Authors' contributions

JSS carried out the following tasks: i) preparation of data, ii) development and implementation of all algorithms used in this work and (iii) wrote the manuscript.

MY carried out the following tasks: i) supervision of the whole work from inception to completion, ii) revised the manuscript and provided critical feedback to improve the intellectual merit of the contribution.

Both the authors read and approved the final manuscript.

## Supplementary Material

Additional file 1 3D star coordinate projection. The basic concepts of star coordinate projection are illustrated. Click here for file

Additional file 2 Differentially expressed genes for Leukemia data. The genes selected by unified framework for the Leukemia data [30]. Click here for file

Additional file 3 Common genes for Leukemia data. The genes found using unified framework common to the genes found by Golub et al [30]. Click here for file

Additional file 4 Differentially expressed genes for Gastric cancer data. The genes selected by unified framework for the Gastric cancer data [29]. Click here for file

Additional file 5 Common genes for Gastric cancer data. The genes found using unified framework common to the genes found by Chen et al [29]. Click here for file

Additional file 6 Differentially expressed genes for Colon cancer data. The genes selected by unified framework for the Colon cancer data [8]. Click here for file

Additional file 7 Differentially expressed genes for Codelink data. The genes selected by unified framework for the Codelink data [31]. Click here for file

Additional file 8 Differentially expressed genes for MAS05 data. The genes selected by unified framework for the MAS05 data [31]. Click here for file

Additional file 9 Differentially expressed genes for dChip data. The genes selected by unified framework for the dChip data [31]. Click here for file
