# Supplementary material for: A unified framework for finding differentially expressed genes from microarray experiments
Source: BMC Bioinformatics. 2007 Sep 18;8:347. doi: 10.1186/1471-2105-8-347 (PMC2099446; doi:10.1186/1471-2105-8-347)
Supplement: Additional file 1 — 3D star coordinate projection. The basic concepts of star coordinate projection are illustrated. [file 1471-2105-8-347-S1.pdf]

3D star coordinate projection

The basic concept of star coordinate projection is first explained using 2DSCP with an example. The extension of 2D SCP to 3D SCP and then to S3D SCP are discussed next in the order mentioned. The subsection ends with an automated method to find the best projection among the possible projections.

2D star coordinate projection

Kandogan introduced the concept of star coordinate projection in 2000 [1]. To illustrate the idea behind 2DSCP, consider an example data point of dimension 8 given by $A = [0.37\ \ 0.43\ \ 0.01\ \ 0.869\ \ 0.06\ \ 0.95\ \ 0.98]$. The dimensional anchors for 2DSCP are shown in Fig. 1. The dimensional anchors multiplied by the dimension values for data point '*A'* are shown in Fig. 2. As shown in Fig. 2, each of the dimensional anchors is scaled to the dimensional values of data point '*A*'. The scaled dimensional anchors for data point '*A*' are connected in order as shown in Fig. 3. The point of destination after covering all the dimensional anchors is the point of representation for the data point '*A*'. As a result, the 8 dimensional data point is now represented in two dimensions. The same process is repeated for all the data points. The dimensional anchors as shown in Fig. 1 may be arranged in multiple ways there by offering multiple ways to project the data. Also, in the above example, it is assumed that all the dimensional anchors are of equal length as shown in Fig. 1. Kandogan proposes use of random lengths of dimensional anchors to assign importance [1]. The different arrangements coupled with different length assignments of dimensional anchors are explored until a pattern is observed in the data. The number of arrangements that need to be explored is not known and hence, as will be shown later, the automated method that provides possible best projections among the possible projections is extremely important for SCP based projections.

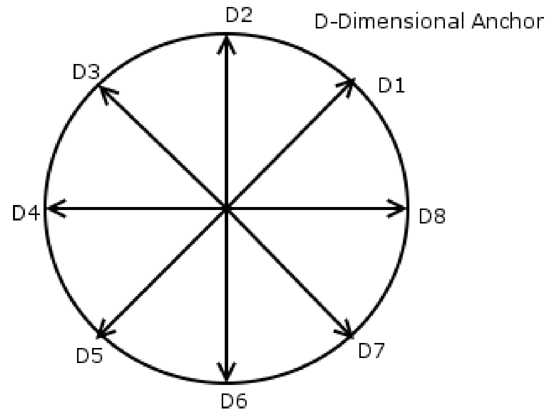

Fig. 1. 2D Star Coordinate

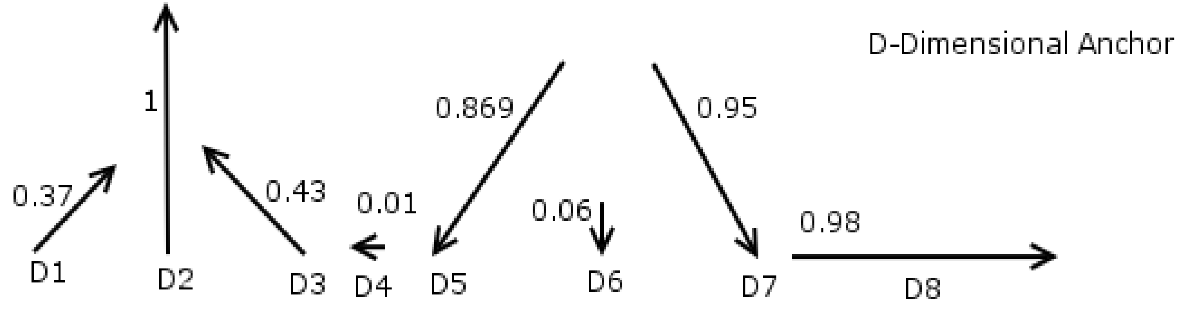

Fig. 2. Dimensional anchors for data point A

3D star coordinate projection

The 3DSCP is an extension of 2DSCP. Instead of the dimensional anchors being arranged along a circle, the dimensional anchors are arranged along a sphere as shown in Fig. 4. This provides more flexibility for arrangement of the dimensional anchors. It also facilitates 3D view of the data. The different steps involved in 3DSCP may be summarized as:

1. Initialization
2. Projection into 3D Space

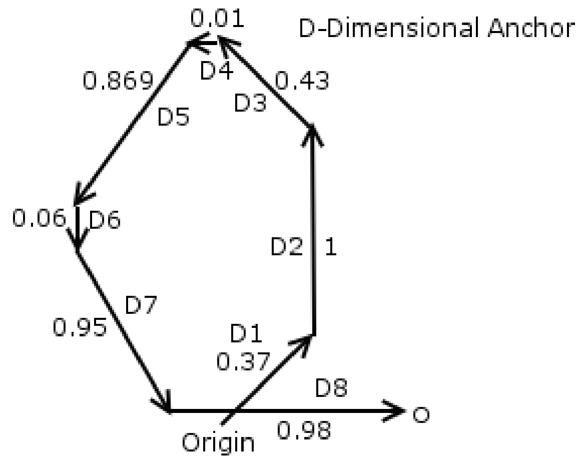

Fig. 3. Projection of data point A

Step1: Initialization

Arrange the dimensional anchors of random length into 3D space at random angles.

Step2: Projection

Let 'N' be the number of data points that need to be visualized and 'n' be the number of attributes (dimensions). The data matrix $D = \begin{bmatrix} d_{11} & d_{12} \ldots & d_{1n} \\ d_{21} & d_{22} \ldots & d_{2n} \\ . & . & . \\ d_{N1} & d_{N2} \ldots & d_{Nn} \end{bmatrix}$ is of dimension $N \times n$ and its elements d_{ij} represent dimension 'j' of data point 'i'. The Fig. 4 shows the 3D star coordinate system. Shaik et al propose resolving the vectors into components for projection [2]. As shown in Fig. 4, the dimensional anchor V is resolved into three components (u_x, u_y, u_z) along 'x', 'y' and 'z' directions. The high dimensional dataset 'D' has 'n' such dimensional anchors radiating into 3D space. The projection of the data point in n-dimensions to 3 dimensions is obtained by summing up the vector components (u_x, u_y, u_z) on each dimensional anchor weighted by its respective attribute value. The components (u_x, u_y, u_z) are given by Eqs. (1-3).

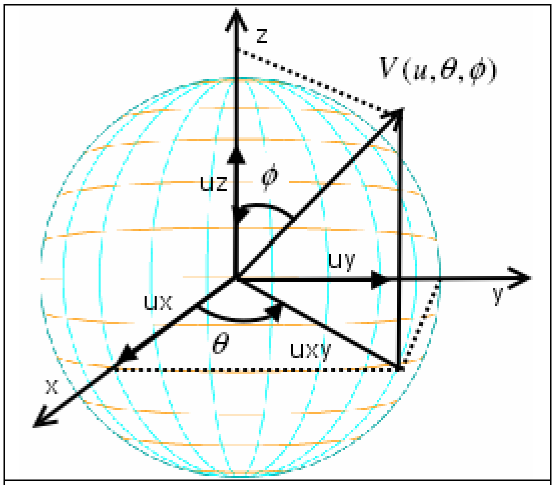

Fig. 4. 3D Star Coordinate System

$$u_x = u \sin\phi \cos\theta, \quad (1)$$

$$u_y = u \sin\phi \sin\theta, \quad (2)$$

$$u_z = u \cos\phi, \quad (3)$$

where, θ and ϕ are the elevation & azimuthal angles and

$$\|u\| = \sqrt{u_x^2 + u_y^2 + u_z^{\ 2}}\ . \qquad (4)$$

The projection of each 'n' dimensional data point 'D_i' is given by $P_j(X,Y,Z) = (X_P, Y_P, Z_P)$ where,

$$\begin{bmatrix} \mathrm{X_p} = O_x + \sum_{i=1}^{n} u_{xi}(d_{j,i} - \min(d_{i,.})), \\ \mathrm{Y_p} = O_y + \sum_{i=1}^{n} u_{yi}(d_{j,i} - \min(d_{i,.})), \\ \mathrm{Z_p} = O_z + \sum_{i=1}^{n} u_{zi}(d_{j,i} - \min(d_{i,.})). \end{bmatrix} \qquad (5)$$

Here, O_x, O_y and O_z are the coordinates defining present origin of the system. The vectors in 'n' dimensional space are therefore projected into 3D space given by $P\ (X,Y,Z)$. It is now easy to visualize various dimensions radiating at random angles in a pseudo sphere, more than one such combination providing insight into the underlying distribution.

1. Kandogan E: **Star Coordinates: A multi-dimensional Visualization Technique with Uniform treatment of Dimensions**. *Proceedings of IEEE information Visualization Symposium (Hot Topics)* 2000:4-8.
2. Shaik J, Yeasin M: **Visualization of High Dimensional Data using an Automated 3D Star Co-ordinate System**. *Proceedings of IEEE IJCNN'06, Vancouver, Canada* 2006:2318-2325.
