# Supplementary material for: A unified framework for finding differentially expressed genes from microarray experiments
Source: BMC Bioinformatics. 2007 Sep 18;8:347. doi: 10.1186/1471-2105-8-347 (PMC2099446; doi:10.1186/1471-2105-8-347)
Supplement: Additional file 2 — Differentially expressed genes for Leukemia data. The genes selected by unified framework for the Leukemia data [30]. [file 1471-2105-8-347-S2.pdf]

Differentially expressed genes for Leukemia dataset

Rank Gene Name
1 CST3 Cystatin C (amyloid angiopathy and cerebral hemorrhage)
2 CYSTATIN A
3 PLECKSTRIN
4 DF D component of complement (adipsin)
5 NF-IL6-beta protein mRNA
6 CD33 CD33 antigen (differentiation antigen)
7 PPBP Connective tissue activation peptide III
8 Azurocidin gene
9 GLUTATHIONE S-TRANSFERASE, MICROSOMAL
10 Epb72 gene exon 1
11 PFC Properdin P factor, complement
12 RNS2 Ribonuclease 2 (eosinophil-derived neurotoxin; EDN)
13 MANB Mannosidase alpha-B (lysosomal)
14 Epican, Alt. Splice 11
15 Pre-B cell enhancing factor (PBEF) mRNA
16 CD36 CD36 antigen (collagen type I receptor, thrombospondin receptor)
17 Zyxin
18 PACE Paired basic amino acid cleaving enzyme (furin, membrane associated
receptor protein)
19 INTERLEUKIN-8 PRECURSOR
20 Phosphotyrosine independent ligand p62 for the Lck SH2 domain mRNA
21 Uridine phosphorylase
22 CTSD Cathepsin D (lysosomal aspartyl protease)
23 FOS-RELATED ANTIGEN 2
24 PRKCD Protein kinase C, delta
25 PTGS2 Prostaglandin-endoperoxide synthase 2 (prostaglandin G/H synthase
and cyclooxygenase)
26 Cyclooxygenase-2 (hCox-2) gene
27 PTX3 Pentaxin-related gene, rapidly induced by IL-1 beta
28 Amphiregulin (AR) gene
29 Cyclooxygenase-2 (hCox-2) gene
30 PEPTIDYL-PROLYL CIS-TRANS ISOMERASE, MITOCHONDRIAL PRECURSOR
31 GB DEF = PTX3 gene promotor region
32 GRO2 GRO2 oncogene
33 Zinc finger transcription factor hEZF (EZF) mRNA
34 CA2 Carbonic anhydrase II
35 Interleukin 8 (IL8) gene
36 Fc-epsilon-receptor gamma-chain mRNA
37 Myeloid progenitor inhibitory factor-1 MPIF-1 mRNA
38 AFFX-HUMTFRR/M11507_M_at (endogenous control)
39 C-myb gene extracted from Human (c-myb) gene, complete primary cds, and
five complete alternatively spliced cds
40 THBS1 Thrombospondin 1
41 SOD2 Superoxide dismutase 2, mitochondrial
42 ALDR1 Aldehyde reductase 1 (low Km aldose reductase)
43 ADM Adrenomedullin
44 HLA-DRB1 Major histocompatibility complex, class II, DR beta 1
45 SELL Leukocyte adhesion protein beta subunit
46 PRG1 Proteoglycan 1, secretory granule
47 IL6 Interleukin 6 (B cell stimulatory factor 2)
48 GB DEF = GPSAT=glycophorin SAT [human, peripheral bloods, mRNA Partial,
407 nt]
49 Inducible protein mRNA

50 SNRPN Small nuclear ribonucleoprotein polypeptide N
51 HOXA9 Homeo box A9
52 Putative cyclin G1 interacting protein mRNA, partial sequence
53 ELA2 Elastatse 2, neutrophil
54 NADPH-flavin reductase
55 CATHEPSIN G PRECURSOR
56 SPTAN1 Spectrin, alpha, non-erythrocytic 1 (alpha-fodrin)
57 LYZ Lysozyme
58 LEUKOCYTE ELASTASE INHIBITOR
59 KIAA0022 gene
60 GB DEF = Selenium-binding protein (hSBP) mRNA
61 PAI2 Plasminogen activator inhibitor, type II (arginine-serpin)
62 Ninjurin1 mRNA
63 ANPEP Alanyl (membrane) aminopeptidase (aminopeptidase N, aminopeptidase
M, microsomal aminopeptidase, CD13)
64 RETINOBLASTOMA BINDING PROTEIN P48
65 CD44 gene (cell surface glycoprotein CD44) extracted from Human hyaluronate receptor (CD44) gene
66 CHRNA7 Cholinergic receptor, nicotinic, alpha polypeptide 7
67 PROTEASOME IOTA CHAIN
68 Serine palmitoyltransferase (LCB2) mRNA, partial cds
69 SLC4A1 Solute carrier family 4, anion exchanger, member 1 (erythrocyte
membrane protein band 3)
70 AFFX-HUMTFRR/M11507_3_at (endogenous control)
71 Prostate differentiation factor mRNA
72 GRN Granulin
73 No cluster in current Unigene and no Genbank entry for U77396 (qualifier
U77396_at)
74 TCF3 Transcription factor 3 (E2A immunoglobulin enhancer binding factors
E12/E47)
75 ALDH2 Aldehyde dehydrogenase 2, mitochondrial
76 Leukotriene C4 synthase (LTC4S) gene
77 TOP2B Topoisomerase (DNA) II beta (180kD)
78 LYN V-yes-1 Yamaguchi sarcoma viral related oncogene homolog
79 Lysozyme gene (EC 3.2.1.17)
80 LEPR Leptin receptor
81 Butyrophilin (BTF5) mRNA
82 ACADM Acyl-Coenzyme A dehydrogenase, C-4 to C-12 straight chain
83 MYL1 Myosin light chain (alkali)
84 IL6 Interleukin 6 (B cell stimulatory factor 2)
85 GB DEF = Neutrophil elastase gene, exon 5
86 Adenosine triphosphatase, calcium
87 G0S2 gene extracted from Human GOS2 gene, 5' flank and cds
88 LGALS3 Lectin, galactoside-binding, soluble, 3 (galectin 3) (NOTE: redefinition of symbol)
89 CTSL Cathepsin L
90 Oncogene Tls/Chop, Fusion Activated
91 ADP-ribosylation factor-like protein 4 mRNA
92 Macmarcks
93 GB DEF = Uroporphyrinogen decarboxylase (URO-D) gene, partial cds
94 GB DEF = Homeodomain protein HoxA9 mRNA
95 ICAM1 Intercellular adhesion molecule 1 (CD54), human rhinovirus receptor
96 ARHG Ras homolog gene family, member G (rho G)
97 INDUCED MYELOID LEUKEMIA CELL DIFFERENTIATION PROTEIN MCL1
98 PI Protease inhibitor 1 (anti-elastase), alpha-1-antitrypsin
99 MACROPHAGE INFLAMMATORY PROTEIN 1-ALPHA PRECURSOR
100 PF4 Platelet factor 4
101 CCND3 Cyclin D3
102 ERYTHROCYTE PLASMA MEMBRANE 50 KD GLYCOPROTEIN

103 Bcl-2 related (Bfl-1) mRNA
104 TNF-related apoptosis inducing ligand TRAIL mRNA
105 GRO3 GRO3 oncogene
106 VIL2 Villin 2 (ezrin)
107 EPB42 Erythrocyte membrane protein band 4.2
108 Oncoprotein 18 (Op18) gene
109 HU-K4 mRNA
110 DDH1 Dihydrodiol dehydrogenase
111 Adrenal-Specific Protein Pg2
112 LYZ Lysozyme
113 APLP2 Amyloid beta (A4) precursor-like protein 2
114 Receptor of retinoic acid
115 Chorionic Somatomammotropin Hormone Cs-5
116 GB DEF = Integral membrane protein (NRAMP1) gene, exon 5
117 CSNK1D Casein kinase 1, delta
118 CLU Clusterin (complement lysis inhibitor; testosterone-repressed prostate
message 2; apolipoprotein J)
119 ATP6C Vacuolar H+ ATPase proton channel subunit
120 AHR AH-receptor
121 Calcyclin
122 GB DEF = Glycophorin Sta (type A) exons 3 and 4, partial
123 HMG2 High-mobility group (nonhistone chromosomal) protein 2
124 PTGER3 Prostaglandin E receptor 3 (subtype EP3) {alternative products}
125 TUBULIN ALPHA-4 CHAIN
126 Breast epithelial antigen BA46 mRNA
127 Transcriptional activator hSNF2b
128 PLGL Plasminogen-like protein
129 Ifp35 gene extracted from Human BRCA1, Rho7 and vatI genes, and ipf35
gene, partial cds
130 TIAL1 TIA1 cytotoxic granule-associated RNA-binding protein-like 1
131 Integrase gene extracted from Human endogenous retrovirus H clone g10.34
integrase and putative envelope protein genes, partial cds
132 HLA CLASS II HISTOCOMPATIBILITY ANTIGEN, DQ(W1.1) BETA CHAIN PRECURSOR
133 PRB2 locus salivary proline-rich protein mRNA, clone cP7
134 HLA-A MHC class I protein HLA-A (HLA-A28,-B40, -Cw3)
135 Major Histocompatibility Complex, Class I, C (Gb:X58536)
136 Natural killer cell receptor (KIR) mRNA
137 Nkat2b mRNA
138 CYTOCHROME P450 IA2
139 GAPD Glyceraldehyde-3-phosphate dehydrogenase
140 ACTB Actin, beta
141 GB DEF = H2B/h gene
142 GB DEF = Transmembrane protein Tmp21-IIex
143 PRSS3 Protease, serine, 3 (trypsin 3)
144 GB DEF = Glutamate dehydrogenase
145 Metallothionein
146 CYTOCHROME P450 IIA6
147 GB DEF = Histone H3 gene
148 KERATIN, TYPE II CYTOSKELETAL 6D
149 MAGE-5a antigen (MAGE5a) gene
150 GB DEF = BTF3 protein homologue gene
151 GB DEF = Histone H2B.1 (H2B) gene
152 PSG7 Pregnancy-specific beta 1-glycoprotein 7
153 HLA-DRB1 Major histocompatibility complex, class II, DR beta 5
154 PRSS1 Protease, serine, 1 (trypsin 1)
155 S26 from Homo sapiens excision and cross link repair protein (ERCC4)
gene, complete genomic sequence./ntype=DNA /annot=exon
156 KERATIN, TYPE II CYTOSKELETAL 6D
157 KERATIN, TYPE II CYTOSKELETAL 6D

158 KERATIN, TYPE II CYTOSKELETAL 6D
159 (clone Hu lambda-17) lambda-like gene
160 Cytochrome P-450 4 gene
161 PRH1 Proline-rich protein HaeIII subfamily 1
162 Chorionic gonadotropin (hcg) beta subunit mRNA
163 Chorionic gonadotropin beta subunit gene
164 HLA CLASS II HISTOCOMPATIBILITY ANTIGEN, DQ(1) BETA CHAIN PRECURSOR
165 Chorionic somatomammotropin CS-1 gene extracted from Human growth hormone
(GH-1 and GH-2) and chorionic somatomammotropin (CS-1, CS-2 and CS-5) genes
166 IFNA21 Interferon, alpha 21
167 Proline-Rich Protein Prb4, Allele
168 Luteinizing Hormone, Beta Subunit
169 Major Histocompatibility Complex, Class I, E (Gb:M21533)
170 Major Histocompatibility Complex, Class I, E (Gb:M20022)
171 Transcription Factor Btf3b
172 PMS8 mRNA (yeast mismatch repair gene PMS1 homologue), partial cds (Cterminal
region)
173 PMS8 mRNA (yeast mismatch repair gene PMS1 homologue), partial cds (Cterminal
region)
174 HLA-A MHC class I protein HLA-A (HLA-A28,-B40, -Cw3)
175 GB DEF = Endogenous retroviral H protease/integrase-derived ORF1 mRNA, and
putative envelope protein mRNA, partial cds
176 GB DEF = Omega light chain protein 14.1 (Ig lambda chain related) gene,
exon 3
177 GB DEF = Omega light chain protein 14.1 (Ig lambda chain related) gene,
exon 3
178 GB DEF = Immunoglobulin-related 14.1 protein mRNA
179 GB DEF = Immunoglobulin-related 14.1 protein mRNA
180 GB DEF = DNA sequence from PAC 151B14 on chromosome 22q12-qter contains
somatostatin receptor subtype 3 (SSTR3), tRNA, ESTs, CpG island and STS
181 ALPHA-CENTRACTIN
182 GB DEF = Bone marrow serine protease gene (medullasin) (leukocyte
neutrophil elastase gene)
183 ZNF183 gene
184 GB DEF = G-protein coupled receptor
185 GB DEF = R kappa B mRNA
186 SCYA4 Small inducible cytokine A4 (homologous to mouse Mip-1b)
187 MYH7 Myosin, heavy polypeptide 7, cardiac muscle, beta
188 JUNB Jun B proto-oncogene
189 RD Radin blood group
190 GABRB1 Gamma-aminobutyric acid (GABA) A receptor, beta 1
191 RAB2 RAB2, member RAS oncogene family
192 APS Prostate specific antigen
193 PRKCB1 Protein kinase C, beta 1
194 Transcription factor E2F like protein [human, mRNA, 2492 nt]
195 Protein tyrosine phosphatase sigma mRNA
196 Estrogen sulfotransferase mRNA
197 GARS Glycyl-tRNA synthetase
198 Homolog of Drosophila enhancer of split m9/m10 mRNA
199 Transcription factor LSF mRNA
200 ARAF1 V-raf murine sarcoma 3611 viral oncogene homolog 1
201 CD19 CD19 antigen
202 GAP43 Growth associated protein 43
203 NPPB Natriuretic peptide precursor B
204 DNA-BINDING PROTEIN A
205 SELL Leukocyte adhesion protein beta subunit

206 AVPR1B Arginine vasopressin receptor 1B
207 Autotaxin mRNA
208 H2K binding factor 2 (KBF2) mRNA
209 mRNA fragment encoding beta-tubulin. (from clone D-beta-1)
210 GB DEF = HH2B/d gene
211 H4/j gene
212 GB DEF = H2B/l gene
213 H2B/j gene
214 HD21 mRNA
215 GB DEF = Chloride channel (putative) 2163bp
216 GB DEF = Chloride channel (putative) 2139bp
217 NGAL gene
218 Ubiquitin hydrolase
219 GB DEF = RagB protein
220 GB DEF = Anion exchange protein
221 GLUL Glutamate-ammonia ligase (glutamine synthase)
222 RPS26 Ribosomal protein S26
223 ZNF37A Zinc finger protein 37a (KOX 21)
224 L2-9 transcript of unrearranged immunoglobulin V(H)5 pseudogene
225 ECRP gene for eosinophil cationic related protein
226 HLA CLASS I HISTOCOMPATIBILITY ANTIGEN, F ALPHA CHAIN PRECURSOR
227 Alpha-tubulin mRNA
228 Clone 61501 defective mariner transposon Hsmar2 mRNA sequence
229 Death domain containing protein CRADD mRNA
230 GB DEF = Immunoglobulin-like transcript-3 mRNA
231 Steroidogenic factor 1 mRNA
232 TRY8 gene (trypsinogen E) extracted from Human germline T-cell receptor
beta chain TCRBV17S1A1T, TCRBV2S1, TCRBV10S1P, TCRBV29S1P, TCRBV19S1P, TCRBV15S1, TCRBV11S1A1T, HVB relic, TCRBV28S1P, TCRBV34S1, TCRBV14S1, TCRBV3S1,
TCRBV4S1A1T, TRY4, TRY5, TRY6, T
233 GB DEF = Mitochondrial trifunctional protein beta subunit mRNA, partial
cds
234 GB DEF = Small GTP binding protein Rab9 mRNA
235 GB DEF = Beta-2 integrin alphaD subunit (ITGAD) gene, exons 25-30, and
partial cds
236 ICH-2 PROTEASE PRECURSOR
237 Uridine diphosphoglucose pyrophosphorylase mRNA
238 Serine kinase (hPAK65) mRNA, partial cds
239 GB DEF = Anti-B cell autoantibody IgM heavy chain variable V-D-J region
(VH4) gene, clone E11, VH4-63 non-productive rearrangement
240 CELLULAR NUCLEIC ACID BINDING PROTEIN
241 Tyrosine kinase receptor p145TRK-B (TRK-B) mRNA
242 FLT3LG Fms-related tyrosine kinase 3 ligand
243 Delta-globin gene extracted from Human beta globin region on chromosome 11
244 GIF=growth inhibitory factor [human, brain, Genomic, 2015 nt]
245 GB DEF = Transducin-like enhancer protein (TLE4) mRNA, 3' end
246 TRANSDUCIN-LIKE ENHANCER PROTEIN 1
247 COL16A1 Alpha-1 type XVI collagen
248 GB DEF = Histone H3.1 (H1F3) gene
249 FCGR2A Fc fragment of IgG, low affinity IIa, receptor for (CD32)
250 PULMONARY SURFACTANT-ASSOCIATED PROTEIN A PRECURSOR
251 AR Androgen receptor (dihydrotestosterone receptor; testicular feminization; spinal and bulbar muscular atrophy; Kennedy disease)
252 Metallothionein I-B gene
253 Skeletal beta-tropomyosin
254 Metallothionein-Ie gene (hMT-Ie)
255 HMR Hormone receptor (growth factor-inducible nuclear protein N10)
256 Regenerating protein I beta

257 GB DEF = Small proline-rich protein 1 (SPRR1A) gene
258 HPR gene (haptoglobin-related protein) extracted from Human haptoglobin
gene (alpha-2 allele)
259 REG1A Regenerating islet-derived 1 alpha (pancreatic stone protein, pancreatic thread protein)
260 (clone 14VS) metallothionein-IG (MT1G) gene
261 Mucin 6, Gastric (Gb:L07518)
262 Sodium Channel 1
263 Tubulin, Beta
264 Olfactory Receptor Or17-24
265 Myosin, Heavy Polypeptide 9, Non-Muscle
266 Tubulin, Beta 2
267 NAK1 mRNA for DNA binding protein
268 GB DEF = Facioscapulohumeral muscular dystrophy (FSHD) gene region, D4Z4
tandem repeat unit
269 ZNF43 Zinc finger protein 43 (HTF6)
270 GYPB Glycophorin B
271 Major Histocompatibility Complex, Class Ii, Dr Beta 2 (Gb:X65561)
272 Homeotic Protein C6, Class I
273 Major Histocompatibility Complex, Class I (Gb:X12432)
274 Major Histocompatibility Complex, Class Ii Beta W52
275 Neurofibromatosis 2 Tumor Suppressor (Gb:L27065)
276 Nadh-Ubiquinone Oxidoreductase, 39 Kda Subunit
277 Mucin 3, Intestinal (Gb:M55406)
278 GB DEF = DNA mismatch repair protein (hMLH1) mRNA, alternatively spliced,
partial cds
279 PDE4C Phosphodiesterase 4C, cAMP-specific (dunce (Drosophila)-homolog
phosphodiesterase E1)
280 Exon 1b; used only in type 2 transcripts from H.sapiens dbi/acbp gene
exon 1 & 2./ntype=DNA /annot=exon
281 GB DEF = Nonmuscle myosin heavy chain IIB gene, promoter region and exon 1
282 DMA gene extracted from H.sapiens DMA, DMB, HLA-Z1, IPP2, LMP2, TAP1,
LMP7, TAP2, DOB, DQB2 and RING8, 9, 13 and 14 genes
283 DMA gene extracted from H.sapiens DMA, DMB, HLA-Z1, IPP2, LMP2, TAP1,
LMP7, TAP2, DOB, DQB2 and RING8, 9, 13 and 14 genes
284 Butyrophilin (BTF4) mRNA
285 GB DEF = Neurofilament triplet L protein mRNA, partial cds
286 Krueppel-related zinc finger protein (H-plk) mRNA
287 GB DEF = Unproductively rearranged Ig mu-chain mRNA V-region (VD), 5' end,
clone mu-3A1A
288 ZNF91 Zinc finger protein 91 (HPF7, HTF10)
289 ZNF91 Zinc finger protein 91 (HPF7, HTF10)
290 Small Nuclear Ribonucleoprotein U1, 1snrp
291 Small Nuclear Ribonucleoprotein U1, 1snrp
292 Zinc Finger Protein (Gb:M88357)
293 Spliceosomal Protein Sap 62
294 Potassium Channel Protein (Gb:Z11585)
295 Mucin 3, Intestinal (Gb:M55405)
296 Mucin 3, Intestinal (Gb:M55405)
297 GB DEF = Alpha satellite and satellite 3 junction DNA sequence
298 Ins(1,3,4,5)P4-binding protein
299 BETA-2-MICROGLOBULIN PRECURSOR
300 AKT2 V-akt murine thymoma viral oncogene homolog 2
