# Supplementary material for: A unified framework for finding differentially expressed genes from microarray experiments
Source: BMC Bioinformatics. 2007 Sep 18;8:347. doi: 10.1186/1471-2105-8-347 (PMC2099446; doi:10.1186/1471-2105-8-347)
Supplement: Additional file 3 — Common genes for Leukemia data. The genes found using unified framework common to the genes found by Golub et al [30]. [file 1471-2105-8-347-S3.pdf]

# Common genes for Leukemia data

1. C-myb
2. PROTEASOME IOTA CHAIN
3. MYL1 Myosin light chain (alkali)
4. TCF3 Transcription factor 3 (E2A immunoglobulin enhancer binding factors E12/E47)
5. Inducible protein mRNA
6. TOP2B Topoisomerase (DNA) II beta (180kD)
7. ACADM Acyl-Coenzyme A dehydrogenase, C-4 to C-12 straight chain
8. Oncoprotein 18 (Op18) gene
9. FAH Fumarylacetoacetate
10. Zyxin
11. Leukotriene C4 synthase (LTC4S) gene
12. LYN V-yes-1 Yamaguchi sarcoma viral related oncogene homolog
13. HOXA9 Homeo box A9
14. CD33 CD33 antigen (differentiation antigen)
15. DF D component of complement (adipsin)
16. LEPR Leptin receptor
17. CST3 Cystatin C (amyloid angiopathy and cerebral hemorrhage)
18. CD44 gene (cell surface glycoprotein CD44) extracted from Human hyaluronate receptor (CD44) gene
19. Interleukin 8 (IL8) gene
20. CTSD Cathepsin D (lysosomal aspartyl protease)
21. LGALS3 Lectin, galactoside-binding, soluble, 3 (galectin 3) (NOTE: redefinition of symbol)
22. MAJOR HISTOCOMPATIBILITY COMPLEX ENHANCER-BINDING PROTEIN MAD3
23. Lysozyme gene (EC 3.2.1.17)
24. PFC Properdin P factor, complement
