# Supplementary material for: A unified framework for finding differentially expressed genes from microarray experiments
Source: BMC Bioinformatics. 2007 Sep 18;8:347. doi: 10.1186/1471-2105-8-347 (PMC2099446; doi:10.1186/1471-2105-8-347)
Supplement: Additional file 4 — Differentially expressed genes for Gastric cancer data. The genes selected by unified framework for the Gastric cancer data [29]. [file 1471-2105-8-347-S4.pdf]

Differentially expressed genes for Gastric cancer data

Rank	Gene	Name
1	GENE5431X	IMAGE:1600221
2	GENE525X	IMAGE:245990
3	GENE3133X	IMAGE:202535
4	GENE1810X	IMAGE:795755
5	GENE1401X	IMAGE:39593
6	GENE1721X	IMAGE:838285
7	GENE5854X	IMAGE:2250736
8	GENE3355X	IMAGE:119289
9	GENE1257X	IMAGE:78353
10	GENE5777X	IMAGE:240183
11	GENE2267X	IMAGE:785707
12	GENE5486X	IMAGE:119290
13	GENE863X	IMAGE:139009
14	GENE6052X	IMAGE:1899312
15	GENE5258X	IMAGE:1900149
16	GENE610X	IMAGE:487948
17	GENE3624X	IMAGE:68637
18	GENE2386X	IMAGE:232586
19	GENE1135X	IMAGE:785690
20	GENE2960X	IMAGE:796123
21	GENE2477X	IMAGE:898035
22	GENE2705X	IMAGE:898221
23	GENE676X	IMAGE:288961
24	GENE63X	IMAGE:244147
25	GENE1960X	IMAGE:1584859
26	GENE5963X	IMAGE:1899496
27	GENE6667X	IMAGE:785585
28	GENE6209X	IMAGE:1663354
29	GENE2947X	IMAGE:140301
30	GENE5049X	IMAGE:1899230
31	GENE3430X	IMAGE:127486
32	GENE4882X	IMAGE:725405
33	GENE1226X	IMAGE:194515
34	GENE2560X	IMAGE:280683
35	GENE3465X	IMAGE:823615
36	GENE5619X	IMAGE:1659533
37	GENE999X	IMAGE:705274
38	GENE2257X	IMAGE:796083
39	GENE744X	IMAGE:795585
40	GENE385X	IMAGE:126887
41	GENE5807X	IMAGE:2067500
42	GENE4403X	IMAGE:745525
43	GENE3454X	IMAGE:50531
44	GENE5895X	IMAGE:2310169
45	GENE5193X	IMAGE:199648
46	GENE1965X	IMAGE:770581
47	GENE2554X	IMAGE:781047
48	GENE2088X	IMAGE:296095
49	GENE2727X	IMAGE:1101643
50	GENE2789X	IMAGE:248256
51	GENE5525X	IMAGE:1593010
52	GENE1508X	IMAGE:362402
53	GENE5769X	IMAGE:2308409
54	GENE494X	IMAGE:27277
55	GENE2041X	IMAGE:297392
56	GENE5627X	IMAGE:1585952
57	GENE1612X	IMAGE:1031963
58	GENE2695X	IMAGE:250654
59	GENE5930X	IMAGE:1554962

60 GENE107X IMAGE:214162
61 GENE6208X IMAGE:1901472
62 GENE5079X IMAGE:1909163
63 GENE1952X IMAGE:144786
64 GENE253X IMAGE:491692
65 GENE959X IMAGE:142788
66 GENE5504X IMAGE:1669672
67 GENE5554X IMAGE:1609752
68 GENE1250X IMAGE:345538
69 GENE1069X IMAGE:840726
70 GENE5559X IMAGE:1571913
71 GENE2443X IMAGE:240648
72 GENE1208X IMAGE:39973
73 GENE4133X IMAGE:281908
74 GENE6471X IMAGE:755238
75 GENE1153X IMAGE:841070
76 GENE2407X IMAGE:265102
77 GENE96X IMAGE:756402
78 GENE3442X IMAGE:131316
79 GENE4008X IMAGE:40031
80 GENE1356X IMAGE:769686
81 GENE1655X IMAGE:511820
82 GENE5866X IMAGE:1893136
83 GENE993X IMAGE:896962
84 GENE874X IMAGE:843049
85 GENE144X IMAGE:191664
86 GENE1428X IMAGE:80948
87 GENE3307X IMAGE:785701
88 GENE1544X IMAGE:788256
89 GENE4102X IMAGE:462603
90 GENE1507X IMAGE:200402
91 GENE1517X IMAGE:294273
92 GENE5074X IMAGE:1536451
93 GENE1619X IMAGE:1457341
94 GENE6560X IMAGE:153505
95 GENE2514X IMAGE:725454
96 GENE1908X IMAGE:188232
97 GENE2103X IMAGE:665379
98 GENE2656X IMAGE:79592
99 GENE5086X IMAGE:126458
100 GENE5450X IMAGE:1639531
101 GENE239X IMAGE:784126
102 GENE1432X IMAGE:346696
103 GENE5567X IMAGE:1540227
104 GENE1794X IMAGE:30114
105 GENE493X IMAGE:322511
106 GENE6602X IMAGE:731095
107 GENE2466X IMAGE:144881
108 GENE1772X IMAGE:772880
109 GENE5565X IMAGE:1555659
110 GENE4423X IMAGE:825076
111 GENE3360X IMAGE:840493
112 GENE4794X IMAGE:1493107
113 GENE434X IMAGE:357465
114 GENE4972X IMAGE:984370
115 GENE2209X IMAGE:491478
116 GENE6202X IMAGE:1660409
117 GENE2886X IMAGE:770192
118 GENE1700X IMAGE:595090
119 GENE1675X IMAGE:275738
120 GENE3057X IMAGE:155768
121 GENE2152X IMAGE:232772
122 GENE732X IMAGE:186682
123 GENE5340X IMAGE:135238

124 GENE5104X IMAGE:1947911
125 GENE1317X IMAGE:247117
126 GENE721X IMAGE:812968
127 GENE1694X IMAGE:1605153
128 GENE4224X IMAGE:399390
129 GENE225X IMAGE:925538
130 GENE1194X IMAGE:955521
131 GENE3638X IMAGE:545749
132 GENE5589X IMAGE:1570427
133 GENE3524X IMAGE:46376
134 GENE4636X IMAGE:162718
135 GENE5637X IMAGE:34031
136 GENE6288X IMAGE:1518845
137 GENE1450X IMAGE:298560
138 GENE4776X IMAGE:489533
139 GENE4814X IMAGE:856115
140 GENE1918X IMAGE:209143
141 GENE4574X IMAGE:258265
142 GENE6661X IMAGE:1572704
143 GENE3022X IMAGE:208413
144 GENE962X IMAGE:215000
145 GENE5484X IMAGE:1550894
146 GENE1098X IMAGE:745689
147 GENE3971X IMAGE:167205
148 GENE5409X IMAGE:2019011
149 GENE2433X IMAGE:1046495
150 GENE592X IMAGE:1568245
151 GENE4993X IMAGE:26883
152 GENE3880X IMAGE:1418621
153 GENE3557X IMAGE:1032056
154 GENE5445X IMAGE:245351
155 GENE4106X IMAGE:145310
156 GENE4259X IMAGE:378433
157 GENE2463X IMAGE:1699107
158 GENE4023X IMAGE:460435
159 GENE5148X IMAGE:1572723
160 GENE795X IMAGE:117806
161 GENE2602X IMAGE:261472
162 GENE2013X IMAGE:1606304
163 GENE2414X IMAGE:857324
164 GENE2360X IMAGE:461761
165 GENE3731X IMAGE:487932
166 GENE1790X IMAGE:154172
167 GENE5058X IMAGE:448093
168 GENE2932X IMAGE:780994
169 GENE3702X IMAGE:814431
170 GENE3379X IMAGE:1415916
171 GENE5959X IMAGE:1754220
172 GENE5848X IMAGE:2244718
173 GENE5602X IMAGE:1435103
174 GENE1906X IMAGE:129585
175 GENE4363X IMAGE:146882
176 GENE6614X IMAGE:784129
177 GENE6673X IMAGE:592592
178 GENE4954X IMAGE:415046
179 GENE1093X IMAGE:322175
180 GENE3039X IMAGE:460487
181 GENE5463X IMAGE:841326
182 GENE985X IMAGE:162778
183 GENE277X IMAGE:504785
184 GENE4540X IMAGE:289653
185 GENE3491X IMAGE:284882
186 GENE5912X IMAGE:1913928
187 GENE2208X IMAGE:195712

188 GENE2590X IMAGE:785542
189 GENE5436X IMAGE:1636181
190 GENE4519X IMAGE:740965
191 GENE6370X *mitoch. cont. IMAGE:1862251
192 GENE5508X IMAGE:512061
193 GENE4502X IMAGE:1583501
194 GENE4265X IMAGE:396358
195 GENE4132X IMAGE:462983
196 GENE6308X IMAGE:1899292
197 GENE5797X IMAGE:1851004
198 GENE29X IMAGE:46694
199 GENE408X IMAGE:51865
200 GENE3040X IMAGE:66534
201 GENE6633X IMAGE:810391
202 GENE6058X IMAGE:1710872
203 GENE4976X IMAGE:471823
204 GENE2245X IMAGE:200396
205 GENE944X IMAGE:49970
206 GENE4394X IMAGE:51275
207 GENE4345X IMAGE:1420830
208 GENE5636X IMAGE:1540436
209 GENE5393X IMAGE:1643566
210 GENE1484X IMAGE:1591477
