# Supplementary material for: A unified framework for finding differentially expressed genes from microarray experiments
Source: BMC Bioinformatics. 2007 Sep 18;8:347. doi: 10.1186/1471-2105-8-347 (PMC2099446; doi:10.1186/1471-2105-8-347)
Supplement: Additional file 5 — Common genes for Gastric cancer data. The genes found using unified framework common to the genes found by Chen et al [29]. [file 1471-2105-8-347-S5.pdf]

# DEGs common to the DEGs found by Chen et al

1 GENE5431X IMAGE:1600221 309490 MACMARCKS macrophage myristoylated alanine-rich C kinase substrate Hs.75061 AA961735

2 GENE525X IMAGE:245990 102953 RNAHP RNA helicase-related protein Hs.8765 N55459

3 GENE3133X IMAGE:202535 117946 MT1G metallothionein 1G Hs.334409 H53339

4 GENE1810X IMAGE:795755 110403 ESTs Hs.98587 AA460313

5 GENE1401X IMAGE:39593 108104 SST somatostatin Hs.12409 R51912

6 GENE1721X IMAGE:838285 109896 LOC51092 CGI-40 protein Hs.33724 AA457484

7 GENE5854X IMAGE:2250736 313415 MGB2 mammaglobin 2 Hs.97644 AI659370

8 GENE3355X IMAGE:119289 119302 **EST Hs.18239 AI732293

9 GENE1257X IMAGE:78353 107373 RNAHP RNA helicase-related protein
Hs.8765
T56221

10 GENE5777X IMAGE:240183 312481 SLC9A1 solute carrier family 9 (sodium/hydrogen exchanger), isoform 1 (antiporter, Na+/H+, amiloride sensitive)
Hs.170222 H89482

11 GENE2267X IMAGE:785707 113048 PRC1 protein regulator of cytokinesis 1 Hs.344037 AA449336

12 GENE5486X IMAGE:119290 309892 CTXL cortical thymocyte receptor (X. laevis CTX) like Hs.112377 T98050

13 GENE863X IMAGE:139009 104992 FN1 fibronectin 1 Hs.287820 R62612

14 GENE6052X IMAGE:1899312 316130 Homo sapiens cDNA FLJ31206 fis, clone KIDNE2003335 Hs.134478 AI289178

15 GENE5258X IMAGE:1900149 308338 SBB103 hypothetical SBBI03 protein Hs.153639 AI299601

16 GENE610X IMAGE:487948 103442 Homo sapiens cDNA: FLJ22566 fis, clone HSI01980 Hs.348294 AA054715

17 GENE3624X IMAGE:68637 120936 Homo sapiens cDNA FLJ31206 fis, clone KIDNE2003335 Hs.134478 T49815

18 GENE2386X IMAGE:232586 113728 **ESTs, Weakly similar to PC4259 ferritin associated protein [H.sapiens] Hs.308154 H73321

19 GENE1135X IMAGE:785690 106614 Homo sapiens mRNA; cDNA DKFZp564N1662 (from clone DKFZp564N1662) Hs.49117 AA449319

20 GENE2960X IMAGE:796123 116920 SMPD3 **sphingomyelin phosphodiesterase 3, neutral membrane (neutral sphingomyelinase II)
Hs.283616
AA460963

21 GENE2477X IMAGE:898035 114260 AA598950

22 GENE2705X IMAGE:898221 115568 NR4A2 nuclear receptor subfamily 4, group A, member 2 Hs.82120 AA598611

23 GENE676X IMAGE:288961 103832 KIAA0618 KIAA0618 gene product Hs.295112 N62712

24 GENE63X IMAGE:244147 100311 BGN biglycan Hs.821 N51018

25 GENE1960X IMAGE:1584859 111341 ESTs Hs.127196 AA939002

26 GENE5963X IMAGE:1899496 314809 AKR1B10 aldo-keto reductase family 1, member B10 (aldose reductase) Hs.116724 AI301329

27 GENE6667X IMAGE:785585 99898 ESTs Hs.98969 AA448934

28 GENE6209X IMAGE:1663354 318284 ESTs Hs.126059 AI129156

29 GENE2947X IMAGE:140301 116870 Homo sapiens cDNA FLJ11041 fis, clone PLACE1004405 Hs.28792 R66924

30 GENE5049X IMAGE:1899230 307038 H.sapiens mRNA for metallothionein isoform 1R Hs.348990 AI289110

31 GENE3430X IMAGE:127486 119803 ESTs Hs.21798 R09053

32 GENE4882X IMAGE:725405 226697 Homo sapiens mRNA; cDNA DKFZp586A0618 (from clone DKFZp586A0618) Hs.349755 AA292086

33 GENE1226X IMAGE:194515 107206 SULT1C1 sulfotransferase family, cytosolic, 1C, member 1 Hs.38084 R86241

34 GENE2560X IMAGE:280683 114757 ESTs Hs.102650 N50517

35 GENE3465X IMAGE:823615 119984 Homo sapiens cDNA: FLJ21245 fis, clone COL01184 Hs.99785 AA496957

36 GENE5619X IMAGE:1659533 310750 MATN4 matrilin 4 Hs.278489 AI038302

37 GENE999X IMAGE:705274 105763 DGKD diacylglycerol kinase, delta (130kD) Hs.115907 AA280691

38 GENE2257X IMAGE:796083 112973 DJ1042K10.2 hypothetical protein Hs.22129 AA460372

39 GENE744X IMAGE:795585 104239 FLJ10160 hypothetical protein FLJ10160 similar to insulin related protein 2 Hs.214397 AA459702

40 GENE385X IMAGE:126887 102248 ESTs Hs.293649 R07321

41 GENE5807X IMAGE:2067500 312808 LDB3 LIM domain binding 3 Hs.49998 AI383171

42 GENE4403X IMAGE:745525 224080 DPT dermatopontin Hs.80552 AA626248

43 GENE3454X IMAGE:50531 119927 H18025

44 GENE5895X IMAGE:2310169 313958 ALDH3A1 aldehyde dehydrogenase 3 family, memberA1 Hs.575 AI652557

45 GENE5193X IMAGE:199648 307914 AKR1C2 aldo-keto reductase family 1, member C2 (dihydrodiol dehydrogenase 2; bile acid binding protein; 3-alpha hydroxysteroid dehydrogenase, type III) Hs.201967 R96609

46 GENE1965X IMAGE:770581 111359 MGC11332 hypothetical protein

MGC11332 Hs.98798 AA434145
47 GENE2554X IMAGE:781047 114729 BUB1 BUB1 budding uninhibited by benzimidazoles 1 homolog (yeast) Hs.98658 AA430092

48 GENE2088X IMAGE:296095 112055 TFDP1 transcription factor Dp-1 Hs.79353 N73611

49 GENE2727X IMAGE:1101643 115680 MUC6 mucin 6, gastric Hs.351428 AA588236

50 GENE2789X IMAGE:248256 116071 KIAA1691 KIAA1691 protein Hs.94761 N58487

51 GENE5525X IMAGE:1593010 310111 KIAA0545 KIAA0545 protein Hs.129943 AA985595

52 GENE1508X IMAGE:362402 108700 LOC57797 hypothetical protein R31240_1 Hs.20707 AA018449

53 GENE5769X IMAGE:2308409 312399 KIAA1077 KIAA1077 protein Hs.70823 AI653116

54 GENE494X IMAGE:27277 102783 ESTs Hs.22627 R19096

55 GENE2041X IMAGE:297392 111826 MT1X **metallothionein 1X Hs.278462 N80129

56 GENE5627X IMAGE:1585952 310794 ESTs Hs.128665 AA974305

57 GENE1612X IMAGE:1031963 109275 Homo sapiens mRNA; cDNA DKFZp586A0618 (from clone DKFZp586A0618) Hs.349755 AA609783

58 GENE2695X IMAGE:250654 115502 SPARC secreted protein, acidic, cysteine-rich (osteonectin) Hs.111779 H95959

59 GENE5930X IMAGE:1554962 314338 ESTs Hs.270149 AA953560

60 GENE107X IMAGE:214162 100560 MT1H metallothionein 1H Hs.2667 H77597

61 GENE6208X IMAGE:1901472 318274 ESTs, Weakly similar to I38022 hypothetical protein [H.sapiens] Hs.150821 AI302661

62 GENE5079X IMAGE:1909163 307226 B3GNT6 UDP-GlcNAc:betaGal beta-1,3-Nacetylglucosaminyltransferase 6 Hs.8526 AI300634

63 GENE1952X IMAGE:144786 111282 BGN biglycan Hs.821 R77226

64 GENE253X IMAGE:491692 101498 COL4A1 collagen, type IV, alpha 1 Hs.119129 AA150402

65 GENE959X IMAGE:142788 105588 SERPINH2 serine (or cysteine) proteinase inhibitor, clade H (heat shock protein 47), member 2 Hs.9930 R71093

66 GENE5504X IMAGE:1669672 309986 THY1 Thy-1 cell surface antigen Hs.125359 AI057267

67 GENE5554X IMAGE:1609752 310303 APOC1 apolipoprotein C-I Hs.268571 AA991590

68 GENE1250X IMAGE:345538 107321 CTSL cathepsin L Hs.78056 W73874

69 GENE1069X IMAGE:840726 106203 FN1 **fibronectin 1 Hs.287820

AA487845

70 GENE5559X IMAGE:1571913 310358 TU3A TU3A protein Hs.8022 AA932696

71 GENE2443X IMAGE:240648 114063 ESTs Hs.41379 H90280

72 GENE1208X IMAGE:39973 107104 EST Hs.25922 R52522

73 GENE4133X IMAGE:281908 222543 Homo sapiens mRNA full length insert cDNA clone EUROIMAGE 1913076 Hs.41271 N51859

74 GENE6471X IMAGE:755238 98759 ABCA5 **ATP-binding cassette, subfamily A (ABC1), member 5 Hs.180513 AA422007

75 GENE1153X IMAGE:841070 106693 YARS tyrosyl-tRNA synthetase Hs.239307 AA486761

76 GENE2407X IMAGE:265102 113827 AIP-1 abl-interactor 12 (SH3-containing protein) Hs.343575 N21334

77 GENE96X IMAGE:756402 100507 AA482115

78 GENE3442X IMAGE:131316 119890 ESTs, Weakly similar to T33068 hypothetical protein C35E7.9 - Caenorhabditis elegans [C.elegans] Hs.339665, R22949
79 GENE4008X IMAGE:40031 221867 FLJ12806 hypothetical protein FLJ12806 Hs.107637 R53342

80 GENE1356X IMAGE:769686 107861 THY1 Thy-1 cell surface antigen Hs.125359 AA428836

81 GENE1655X IMAGE:511820 109517 FLJ20871 hypothetical protein FLJ20871 similar to FSP27 Hs.301002 AA088748

82 GENE5866X IMAGE:1893136 313617 ESTs Hs.145968 AI278518

83 GENE993X IMAGE:896962 105740 ACADS acyl-Coenzyme A dehydrogenase, C-2 to C-3 short chain Hs.348900 AA676663

84 GENE874X IMAGE:843049 105078 MCM4 MCM4 minichromosome maintenance deficient 4 (S. cerevisiae) Hs.154443 AA485983

85 GENE144X IMAGE:191664 100797 THBS2 thrombospondin 2 Hs.108623 H38013

86 GENE1428X IMAGE:80948 108303 SLU7 step II splicing factor SLU7 Hs.76325 T70057

87 GENE3307X IMAGE:785701 119005 RAB31 RAB31, member RAS oncogene family Hs.223025 AA449333

88 GENE1544X IMAGE:788256 108920 KNSL5 kinesin-like 5 (mitotic kinesin-like protein 1) Hs.270845 AA452513

89 GENE4102X IMAGE:462603 222350 ESTs Hs.93102 AA704965

90 GENE1507X IMAGE:200402 108697 C20orf129 chromosome 20 open reading frame 129 Hs.70704 R96941

91 GENE1517X IMAGE:294273 108783 PXMP2 peroxisomal membrane protein 2 (22kD) Hs.49912 N70714

92 GENE5074X IMAGE:1536451 307205 ESTs Hs.126714 AA919126

93 GENE1619X IMAGE:1457341 109290 CA1 carbonic anhydrase I Hs.23118 AA911903

94 GENE6560X IMAGE:153505 99237 DPT dermatopontin Hs.80552 R48303

95 GENE2514X IMAGE:725454 114475 CKS2 CDC28 protein kinase 2 Hs.83758 AA292964

96 GENE1908X IMAGE:188232 110999 KLF4 Kruppel-like factor 4 (gut) Hs.7934 H45668

97 GENE2103X IMAGE:665379 112170 FLJ22174 hypothetical protein FLJ22174 Hs.7734 AA194807

98 GENE2656X IMAGE:79592 115249 AKR7A2 aldo-keto reductase family 7, memberA2 (aflatoxin aldehyde reductase) Hs.6980 T62715

99 GENE5086X IMAGE:126458 307281 ESTs, Moderately similar to AF078844 1 hqp0376 protein [H.sapiens] Hs.188518 R06601

100 GENE5450X IMAGE:1639531 309617 RAB27A RAB27A, member RAS oncogene family Hs.50477 AI032392

101 GENE239X IMAGE:784126 101392 TST thiosulfate sulfurtransferase (rhodanese) Hs.351863 AA432063

102 GENE1432X IMAGE:346696 108327 TEAD4 TEA domain family member 4 Hs.94865 W72159

103 GENE5567X IMAGE:1540227 310388 C20orf1 chromosome 20 open reading frame 1 Hs.9329 AA936183

104 GENE1794X IMAGE:30114 110325 DKFZP564F013 **hypothetical protein DKFZp564F013 Hs.128653 R14908

105 GENE493X IMAGE:322511 102776 Homo sapiens mRNA; cDNA DKFZp564D1462 (from clone DKFZp564D1462) Hs.85335 W15339

106 GENE6602X IMAGE:731095 99493 NUF2R hypothetical protein NUF2R Hs.234545 AA421171

107 GENE2466X IMAGE:144881 114204 CALU calumenin Hs.7753 R78585

108 GENE1772X IMAGE:772880 110219 Homo sapiens hypothetical protein FLJ21127 (FLJ21127), mRNA Hs.351440 AA428455

109 GENE5565X IMAGE:1555659 310380 GPX3 glutathione peroxidase 3 (plasma) Hs.336920 AI147534

110 GENE4423X IMAGE:825076 224164 APT6M8-9 **ATPase, H+ transporting, lysosomal (vacuolar proton pump) membrane sector associated protein M8-9 Hs.183434 AA489232

111 GENE3360X IMAGE:840493 119331 RNASE1 ribonuclease, RNase A family, 1 (pancreatic) Hs.78224 AA485893

112 GENE4794X IMAGE:1493107 226217 ESTs, Highly similar to LB4D_HUMAN NADP-DEPENDENT LEUKOTRIENE B4 12-HYDROXYDEHYDROGENASE [H.sapiens] Hs.348909 AA876375

113 GENE434X IMAGE:357465 102522 DMRT2 doublesex and mab-3 related transcription factor 2 Hs.59506 W93861

114 GENE4972X IMAGE:984370 229002 PSCA prostate stem cell antigen

Hs.20166 AA525838

115 GENE6202X IMAGE:1660409 318234 Human DNA sequence from cDNA 16pHQG;19 from chromosome 16p13.3 Hs.306284 AI041729

116 GENE2886X IMAGE:770192 116580 LGALS9 lectin, galactoside-binding, soluble, 9 (galectin 9) Hs.81337 AA434102

117 GENE1700X IMAGE:595090 109796 ESTs, Weakly similar to AC004858 3 U1 small ribonucleoprotein 1SNRP homolog [H.sapiens] Hs.59507 AA173907

118 GENE3057X IMAGE:155768 117498 Homo sapiens, clone IMAGE:4296901, mRNA Hs.350038 R72097

119 GENE2152X IMAGE:232772 112450 ESTs, Highly similar to SMHU1B metallothionein 1B [H.sapiens] Hs.36102 H72722

120 GENE732X IMAGE:186682 104158 TFF1 trefoil factor 1 (breast cancer, estrogen-inducible sequence expressed in) Hs.350470 R83377

121 GENE5340X IMAGE:135238 308937 ESTs Hs.161049 R32738

122 GENE5104X IMAGE:1947911 307379 TU3A TU3A protein Hs.8022 AI350508

123 GENE1317X IMAGE:247117 107685 AGXT alanine-glyoxylate aminotransferase (oxalosis I; hyperoxaluria I; glycolicaciduria; serine-pyruvate aminotransferase) Hs.144567 N57872

124 GENE721X IMAGE:812968 104107 PTPRN2 protein tyrosine phosphatase, receptor type, N polypeptide 2 Hs.74624 AA464590

125 GENE1694X IMAGE:1605153 109763 ESTs Hs.129136 AA988036

126 GENE4224X IMAGE:399390 223015 LTBP4 latent transforming growth factor beta binding protein 4 Hs.85087 AA732832

127 GENE225X IMAGE:925538 101331 AA534286

128 GENE1194X IMAGE:955521 107021 ESTs Hs.165337 AA506672

129 GENE3638X IMAGE:545749 121269 BCAS1 breast carcinoma amplified sequence 1 Hs.129057 AA079342

130 GENE5589X IMAGE:1570427 310530 MGC4309 hypothetical protein MGC4309 Hs.32417 AA931898

131 GENE5637X IMAGE:34031 310927 EST Hs.302343 R44730

132 GENE6288X IMAGE:1518845 319362 ESTs Hs.20726 AA911063

133 GENE1450X IMAGE:298560 108402 AKR1C2 **aldo-keto reductase family 1, member C2 (dihydrodiol dehydrogenase 2; bile acid binding protein; 3-alpha hydroxysteroid dehydrogenase, type III) Hs.201967 N74260

134 GENE4776X IMAGE:489533 226135 LOC51706 cytochrome b5 reductase 1 (B5R.1) Hs.289113 AA098866

135 GENE4814X IMAGE:856115 226312 PSCA prostate stem cell antigen Hs.20166 AA630584

136 GENE1918X IMAGE:209143 111044 ALDH6A1 aldehyde dehydrogenase 6 family, member A1 Hs.293970 H63534

137 GENE4574X IMAGE:258265 225052 PKIB protein kinase (cAMP-dependent,

catalytic) inhibitor beta Hs.106106 N30669

138 GENE6661X IMAGE:1572704 99870 ESTs Hs.128002 AA972545

139 GENE3022X IMAGE:208413 117245 HPN hepsin (transmembrane protease, serine 1) Hs.823 H62162

140 GENE962X IMAGE:215000 105603 VIPR1 vasoactive intestinal peptide receptor 1 Hs.348500 H73241

141 GENE5484X IMAGE:1550894 309880 ESTs, Highly similar to A46297 beta-1,6-N-acetylglucosaminyltransferase [H.sapiens] Hs.116346 AA913127

142 GENE1098X IMAGE:745689 106374 ESTs, Highly similar to LB4D_HUMAN NADP-DEPENDENT LEUKOTRIENE B4 12-HYDROXYDEHYDROGENASE [H.sapiens] Hs.348909 AA420455

143 GENE3971X IMAGE:167205 221649 SELENBP1 selenium binding protein 1 Hs.334841 R90933

144 GENE5409X IMAGE:2019011 309373 MT3 metallothionein 3 (growth inhibitory factor (neurotrophic)) Hs.73133 AI362950

145 GENE2433X IMAGE:1046495 114007 EST Hs.291183 AA621138

146 GENE592X IMAGE:1568245 103323 ESTs Hs.81295 AA928869

147 GENE4993X IMAGE:26883

148 GENE3880X IMAGE:1418621 221061 HSY11339 GalNAc alpha-2, 6-sialyltransferase I, long form Hs.105352 AA878120

149 GENE3557X IMAGE:1032056 120551 EST Hs.112806 AA610043

150 GENE5445X IMAGE:245351 309576 EST Hs.161467 N53470

151 GENE4106X IMAGE:145310 222382 PRSS12 protease, serine, 12 (neurotrypsin, motopsin) Hs.22404 R77783

152 GENE4259X IMAGE:378433 223251 FLJ11323 hypothetical protein FLJ11323 Hs.25625 AA775600

153 GENE2463X IMAGE:1699107 114195 ESTs Hs.130308 AI003513

154 GENE4023X IMAGE:460435 221982 ESTs Hs.117067 AA677561

155 GENE5148X IMAGE:1572723 307615 Homo sapiens, Similar to RIKEN cDNA 1700010L19 gene, clone MGC:16214 IMAGE:3659061, mRNA, complete cds Hs.103441 AA969822

156 GENE795X IMAGE:117806 104520 SLU7 step II splicing factor SLU7 Hs.76325 T90492

157 GENE2602X IMAGE:261472 114954 ORF1-FL49 putative nuclear protein ORF1-FL49 Hs.323512 H99035

158 GENE2013X IMAGE:1606304 111709 ESTs Hs.129220 AA991182

159 GENE2414X IMAGE:857324 113859 ESTs, Weakly similar to ALU1_HUMAN ALU SUBFAMILY J SEQUENCE CONTAMINATION WARNING ENTRY [H.sapiens] Hs.208385 AA668698

160 GENE2360X IMAGE:461761 113576 ANG angiogenin, ribonuclease, RNase A family, 5 Hs.332764 AA682399

161 GENE3731X IMAGE:487932 220140 SYTL2 synaptotagmin-like 2 Hs.92254 AA045284

162 GENE1790X IMAGE:154172 110304 FCGBP Fc fragment of IgG binding protein Hs.111732 R52030

163 GENE5058X IMAGE:448093 307080 Homo sapiens cDNA FLJ31317 fis, clone LIVER1000421, moderately similar to CYTOCHROME P450 3A5 (EC 1.14.14.1) Hs.350826 AA702706

164 GENE2932X IMAGE:780994 116790 KIAA1543 KIAA1543 protein Hs.17686 AA445996

165 GENE3702X IMAGE:814431 186064 ESTs Hs.95898 AA458945

166 GENE3379X IMAGE:1415916 119494 ESTs Hs.126909 AA826513

167 GENE5959X IMAGE:1754220 314744 Homo sapiens cDNA FLJ20763 fis, clone COL09911 Hs.299329 AI204285

168 GENE5848X IMAGE:2244718 313344 GCNT1 glucosaminyl (N-acetyl) transferase 1, core 2 (beta-1,6-N-acetylglucosaminyltransferase) Hs.159642 AI657057

169 GENE5602X IMAGE:1435103 310591 ATP2A3 ATPase, Ca++ transporting, ubiquitous Hs.5541 AA857542

170 GENE1906X IMAGE:129585 110993 EST, Moderately similar to Cd-7 Metallothionein-2 [H.sapiens] Hs.223649 R16539

171 GENE4363X IMAGE:146882 223871 UBE2C ubiquitin-conjugating enzyme E2C Hs.93002 R80790

172 GENE6614X IMAGE:784129 99545 YWHAG tyrosine 3-monooxygenase/tryptophan 5-monooxygenase activation protein, gamma polypeptide Hs.25001 AA432085

173 GENE6673X IMAGE:592592 99925 MUC5AC mucin 5, subtypes A and C, tracheobronchial/gastric Hs.103707 AA159552

174 GENE4954X IMAGE:415046 227050 FLJ22795 hypothetical protein FLJ22795 Hs.288390 W93108

175 GENE1093X IMAGE:322175 106311 Homo sapiens cDNA FLJ33142 fis, clone UTERU1000192 Hs.351466 W37777

176 GENE3039X IMAGE:460487 117363 LTF lactotransferrin Hs.105938 AA677706

177 GENE5463X IMAGE:841326 309702 ESTs Hs.162082 AA487424

178 GENE985X IMAGE:162778 105714 EST Hs.32347 H27560

179 GENE277X IMAGE:504785 101650 VAPB VAMP (vesicle-associated membrane protein)-associated protein B and C Hs.182625 AA148401

180 GENE5912X IMAGE:1913928 314149 ESTs Hs.165337 AI309109

181 GENE2208X IMAGE:195712 112778 CYP2C9 cytochrome P450, subfamily IIC (mephenytoin 4-hydroxylase), polypeptide 9 Hs.167529 R89491

182 GENE2590X IMAGE:785542 114906 Homo sapiens cDNA FLJ31968 fis, clone NT2RP7007975, highly similar to Homo sapiens BAC526N18 neurexin III gene

Hs.247837 AA450335
183 GENE5436X IMAGE:1636181 309530 SCYB14 small inducible cytokine subfamily B (Cys-X-Cys), member 14 (BRAK) Hs.24395 AI016051

184 GENE4519X IMAGE:740965 224684 LCE hypothetical protein MGC5487 Hs.211556 AA478315

185 GENE6370X *mitoch. cont. IMAGE:1862251 433332 *mitoch. cont.

186 GENE5508X IMAGE:512061 310001 AA128199

187 GENE4502X IMAGE:1583501 224583 DJ1042K10.2 hypothetical protein Hs.22129 AA971563

188 GENE4265X IMAGE:396358 223274 ESTs Hs.121574 AA758379

189 GENE4132X IMAGE:462983 222538 RECQL5 RecQ protein-like 5 Hs.33818 AA682447

190 GENE6308X IMAGE:1899292 319710 KIAA0828 KIAA0828 protein Hs.4984 AI289160

191 GENE5797X IMAGE:1851004 312728 KIAA1524 KIAA1524 protein Hs.151343 AI248987

192 GENE29X IMAGE:46694 100151 FLJ12666 hypothetical protein FLJ12666 Hs.23767 H10192

193 GENE408X IMAGE:51865 102388 CA2 carbonic anhydrase II Hs.155097 H23187

194 GENE3040X IMAGE:66534 117378 GCKR glucokinase (hexokinase 4) regulatory protein Hs.89771 T67006

195 GENE6633X IMAGE:810391 99665 HYAL1 hyaluronoglucosaminidase 1 Hs.75619 AA464196

196 GENE6058X IMAGE:1710872 316250 CBX3 chromobox homolog 3 (HP1 gamma homolog, Drosophila) Hs.278554 AI139106

197 GENE4976X IMAGE:471823 236906 Homo sapiens clone 23763 unknown mRNA, partial cds Hs.168694 AA035644

198 GENE2245X IMAGE:200396 112936 ESTs Hs.351268 R97219

199 GENE944X IMAGE:49970 105503 SSB Sjogren syndrome antigen B (autoantigen La) Hs.83715 H29484

200 GENE4394X IMAGE:51275 224012 FLJ20005 hypothetical protein FLJ20005 Hs.184634 H18864

201 GENE4345X IMAGE:1420830 223769 Homo sapiens cDNA FLJ32320 fis, clone PROST2003537 Hs.165464 AA826324

202 GENE5636X IMAGE:1540436 310917 RAB27A RAB27A, member RAS oncogene family Hs.50477 AA927187

203 GENE5393X IMAGE:1643566 309238 CA9 carbonic anhydrase IX Hs.63287 AI023541

204 GENE1484X IMAGE:1591477 108595 CA9 carbonic anhydrase IX Hs.63287 AA976371
