# Supplementary material for: A unified framework for finding differentially expressed genes from microarray experiments
Source: BMC Bioinformatics. 2007 Sep 18;8:347. doi: 10.1186/1471-2105-8-347 (PMC2099446; doi:10.1186/1471-2105-8-347)
Supplement: Additional file 6 — Differentially expressed genes for Colon cancer data. The genes selected by unified framework for the Colon cancer data [8]. [file 1471-2105-8-347-S6.pdf]

Differentially expressed genes for Colon cancer data

1 Hsa.8125 T71025 3' UTR 1 84103 Human (HUMAN);.
2 Hsa.2361 T51534 3' UTR 1 72396 CYSTATIN C PRECURSOR
(HUMAN).
3 Hsa.558 R34698 3' UTR 1 136738 INTERFERON-INDUCIBLE PROTEIN 9-27
(HUMAN);.
4 Hsa.692 M76378 gene 1 "Human cysteine-rich protein (CRP)
gene, exons 5 and 6.
"
5 Hsa.8147 M63391 gene 1 "Human desmin gene,
complete cds.
"
6 Hsa.692 M76378 gene 1 "Human cysteine-rich protein (CRP)
gene, exons 5 and 6.
"
7 Hsa.43279 H64489 3' UTR 2a 238846 LEUKOCYTE ANTIGEN CD37
(Homo sapiens)
8 Hsa.2795 X15183 gene 1 Human mRNA for 90-kDa
heat-shock protein.
9 Hsa.2126 D26129 gene 1 RIBONUCLEASE PANCREATIC
PRECURSOR (HUMAN);contains element MER21 repetitive element ;.
10 Hsa.821 X14958 gene 1 Human hmgI mRNA for high mobility
group protein Y.
11 Hsa.36689 Z50753 gene 1 H.sapiens mRNA for GCAPII/
uroguanylin precursor.
12 Hsa.3152 D31885 gene 1 "Human mRNA (KIAA0069) for
ORF (novel proetin), partial cds.
"
13 Hsa.24506 R44418 3' UTR 2a 34853 EBNA-2 NUCLEAR PROTEIN
(Epstein-barr virus)
14 Hsa.879 H41129 3' UTR 1 175539 GALECTIN-1 (HUMAN);contains Alu
repetitive element;.
15 Hsa.37937 R87126 3' UTR 2a 197371 "MYOSIN HEAVY CHAIN,
NONMUSCLE (Gallus gallus)
"
16 Hsa.831 M22382 gene 1 MITOCHONDRIAL MATRIX PROTEIN P1
PRECURSOR (HUMAN);.
17 Hsa.36665 U31215 gene 1 "Human metabotropic
glutamate receptor 1 alpha (mGluR1alpha) mRNA, complete cds.
"
18 Hsa.8052 T48014 3' UTR 1 72078 HEMOGLOBIN ALPHA CHAIN
(HUMAN).
19 Hsa.3306 X12671 gene 1 Human gene for
heterogeneous nuclear ribonucleoprotein (hnRNP) core protein A1.
20 Hsa.2646 X68277 gene 1 H.sapiens CL 100 mRNA for
protein tyrosine phosphatase.
21 Hsa.3305 X12369 gene 1 "TROPOMYOSIN ALPHA CHAIN,
SMOOTH MUSCLE (HUMAN);.
"
22 Hsa.692 M76378 gene 1 "Human cysteine-rich protein (CRP)
gene, exons 5 and 6.
"
23 Hsa.773 H40095 3' UTR 1 175181 MACROPHAGE MIGRATION INHIBITORY
FACTOR (HUMAN);.
24 Hsa.2863 X70326 gene 1 H.sapiens MacMarcks mRNA.
25 Hsa.3349 X15882 gene 1 Human mRNA for collagen VI
alpha-2 C-terminal globular domain.
26 Hsa.316 M94132 gene 1 Human mucin 2 (MUC2) mRNA
sequence.
27 Hsa.41280 Z49269 gene 1 H.sapiens gene for
chemokine HCC-1.

28 Hsa.1131 T92451 3' UTR 1 118219 "TROPOMYOSIN, FIBROBLAST
AND EPITHELIAL MUSCLE-TYPE (HUMAN);.
"
29 Hsa.41280 Z49269 gene 1 H.sapiens gene for
chemokine HCC-1.
30 Hsa.8374 T59162 3' UTR 2a 74635 SELENIUM-BINDING PROTEIN
(Mus musculus)
31 Hsa.36952 H43887 3' UTR 2a 183264 COMPLEMENT FACTOR D
PRECURSOR (Homo sapiens)
32 Hsa.3068 X16356 gene 1 Human mRNA for
transmembrane carcinoembryonic antigen BGPC (part.) (formerly TM3-CEA).
33 Hsa.3331 T86473 3' UTR 1 114645 NUCLEOSIDE DIPHOSPHATE
KINASE A (HUMAN);.
34 Hsa.549 R36977 3' UTR 1 26045 P03001 TRANSCRIPTION FACTOR IIIA
;.
35 Hsa.579 M80815 gene 1 "H.sapiens a-L-fucosidase gene,
exon 7 and 8, and complete cds.
"
36 Hsa.41338 D31716 gene 1 "Human mRNA for GC box
bindig protein, complete cds.
"
37 Hsa.3648 T56690 3' UTR 2a 69419 CHLORINE CHANNEL PROTEIN
P64 (Bos taurus)
38 Hsa.2553 X74295 gene 1 H.sapiens mRNA for alpha
7B integrin.
39 Hsa.5211 R67358 3' UTR 2a 141200 MAP KINASE PHOSPHATASE-1
(Homo sapiens)
40 Hsa.341 M26683 gene 1 Human interferon gamma treatment
inducible mRNA.
41 Hsa.2967 X75208 gene 1 H.sapiens HEK2 mRNA for
protein tyrosine kinase receptor.
42 Hsa.449 H58397 3' UTR 1 205866 "TRANS-1,2-DIHYDROBENZENE-1,2-DIOL
DEHYDROGENASE (HUMAN);.
"
43 Hsa.1902 L05144 gene 1 "PHOSPHOENOLPYRUVATE
CARBOXYKINASE, CYTOSOLIC (HUMAN);contains Alu repetitive
element;contains
element PTR5 repetitive element ;.
"
44 Hsa.2715 H77597 3' UTR 1 214162 H.sapiens mRNA for
metallothionein (HUMAN);.
45 Hsa.31630 R64115 3' UTR 2a 139618 ADENOSYLHOMOCYSTEINASE
(Homo sapiens)
46 Hsa.1832 J02854 gene 1 "MYOSIN REGULATORY LIGHT
CHAIN 2, SMOOTH MUSCLE ISOFORM (HUMAN);contains element TAR1 repetitive
element
;.
"
47 Hsa.2344 X86693 gene 1 H.sapiens mRNA for hevin
like protein.
48 Hsa.43405 H81558 3' UTR 2a 238704 PROCYCLIC FORM SPECIFIC
POLYPEPTIDE B1-ALPHA PRECURSOR (Trypanosoma brucei brucei)
49 Hsa.9994 T51539 3' UTR 2a 72395 HEPATOCYTE GROWTH FACTORLIKE
PROTEIN PRECURSOR (Homo sapiens) --68dd_5699-4733_4d62-1290_1927
ContentType:
application/octet-stream Content-Transfer-Encoding: 7bit Content-MD5:
NAlwVJQ4msWkg7LKaY/Asw== Content-Description: rank2b.info
50 Hsa.2097 M36634 gene 1 "Human vasoactive
intestinal peptide (VIP) mRNA, complete cds.
"
51 Hsa.1240 M31994 gene 1 "Human cytosolic aldehyde
dehydrogenase (ALDH1) gene, exon 13.
"
52 Hsa.44350 H79136 3' UTR 2a 235022 ALPHA-2-MACROGLOBULIN

PRECURSOR (Homo sapiens)
53 Hsa.14069 T67077 3' UTR 2a 66563 SODIUM/POTASSIUMTRANSPORTING ATPASE GAMMA CHAIN (Ovis aries)
54 Hsa.6904 T48692 3' UTR 2a 69935 ALPHA-2A ADRENERGIC RECEPTOR (Homo sapiens)
55 Hsa.230 U05291 gene 1 "Human fibromodulin mRNA, partial cds.
"
56 Hsa.8223 T94350 3' UTR 2a 119711 PERIPHERAL MYELIN PROTEIN 22 (Homo sapiens)
57 Hsa.601 J05032 gene 1 "Human aspartyl-tRNA synthetase alpha-2 subunit mRNA, complete cds.
"
58 Hsa.6814 H08393 3' UTR 2a 45395 COLLAGEN ALPHA 2(XI) CHAIN (Homo sapiens)
59 Hsa.2291 H06524 3' UTR 1 44386 "GELSOLIN PRECURSOR, PLASMA (HUMAN);.
"
60 Hsa.25322 R44301 3' UTR 2a 34262 MINERALOCORTICOID RECEPTOR (Homo sapiens)
61 Hsa.2456 U25138 gene 1 "Human MaxiK potassium channel beta subunit mRNA, complete cds.
"
62 Hsa.466 U19969 gene 1 "Human two-handed zinc finger protein ZEB mRNA, partial cds.
"
63 Hsa.72 D29808 gene 1 "Human mRNA for T-cell acute lymphoblastic leukemia associated antigen 1 (TALLA-1), complete cds.
"
64 Hsa.11616 T60778 3' UTR 2a 76539 MATRIX GLA-PROTEIN PRECURSOR (Rattus norvegicus)
65 Hsa.33 M64110 gene 1 "Human caldesmon mRNA, complete cds.
"
66 Hsa.41260 L11706 gene 1 "Human hormone-sensitive lipase (LIPE) gene, complete cds.
"
