# Supplementary material for: A unified framework for finding differentially expressed genes from microarray experiments
Source: BMC Bioinformatics. 2007 Sep 18;8:347. doi: 10.1186/1471-2105-8-347 (PMC2099446; doi:10.1186/1471-2105-8-347)
Supplement: Additional file 8 — Differentially expressed genes for MAS05 data. The genes selected by unified framework for the MAS05 data [31]. [file 1471-2105-8-347-S8.pdf]

Differentially expressed genes for MAS05 data

Ser	ind	Genename/function
1	458	Incl X84037:M.musculus mRNA for E-selectin ligand-1 /cds=(6,3533) /gb=X84037 /gi=673435 /ug=Mm.488 /len=3887
2	1931	Incl L41495:Mus musculus protein-serine/threonine kinase (pim-2) mRNA, complete cds /cds=UNKNOWN /gb=L4149
3	1983	Incl AI840329:UI-M-AJ0-abd-b-03-0-UI.s2 Mus musculus cDNA, 3 end /clone=UI-M-AJ0-abd-b-03-0-UI /clone_end=3 /g
4	2336	Incl AF059583:Mus musculus docking protein Dok-R mRNA, complete cds /cds=(43,1281) /gb=AF059583 /gi=308859
5	2937	Incl U15562:Cell division cycle control protein 25C /cds=(319,1662) /gb=U15562 /gi=882119 /ug=Mm.16857 /len=190
6	3372	7748 Cluster Incl AI848367:UI-M-AH1-ago-e-07-0-UI.s1 Mus musculus cDNA, 3 end /clone=UI-M-AH1-ago-e-07-0-UI /clone_end=
7	3925	949.3 Cluster Incl AA656014:vs47g02.r1 Mus musculus cDNA, 5 end /clone=IMAGE-1149458 /clone_end=5 /gb=AA656014 /gi=25
8	4042	Incl AW045279:UI-M-BH1-akn-c-08-0-UI.s1 Mus musculus cDNA, 3 end /clone=UI-M-BH1-akn-c-08-0-UI /clone_end
9	6953	Incl AI840815:UI-M-AH0-adb-c-03-0-UI.s1 Mus musculus cDNA, 3 end /clone=UI-M-AH0-adb-c-03-0-UI /clone_end=
10	7731	Incl U10406:Capping protein beta 1 /cds=(47,880) /gb=U10406 /gi=500746 /ug=Mm.2945 /len=1647
11	8258	Incl AI840972:UI-M-AK0-adk-e-04-0-UI.s1 Mus musculus cDNA, 3 end /clone=UI-M-AK0-adk-e-04-0-UI /clone_end=3
12	11032	3325.1 Cluster Incl AI882416:uc01c09.r1 Mus musculus cDNA, 5 end /clone=IMAGE-1396720 /clone_end=5 /gb=AI882416 /gi=556
13	12047	3153.8 Cluster Incl X70764:ELKL motif kinase /cds=(93,2417) /gb=X70764 /gi=57919 /ug=Mm.4082 /len=2763
14	12103	Incl AI835443:UI-M-AQ0-aad-c-02-0-UI.s1 Mus musculus cDNA, 3 end /clone=UI-M-AQ0-aad-c-02-0-UI /clone_end=
