# Supplementary material for: A unified framework for finding differentially expressed genes from microarray experiments
Source: BMC Bioinformatics. 2007 Sep 18;8:347. doi: 10.1186/1471-2105-8-347 (PMC2099446; doi:10.1186/1471-2105-8-347)
Supplement: Additional file 9 — Differentially expressed genes for dChip data. The genes selected by unified framework for the dChip data [31]. [file 1471-2105-8-347-S9.pdf]

Differentially expressed genes dChip data

Ser ind Gene Name/Function
1 92 Cluster Incl D87990:House mouse; Musculus domesticus mRNA for UDP-galactose transporter related isozyme 1, complete cds /cds=(88,1056) /gb=D87990 /gi
2 363 Incl X02463:Mouse germline immunoglobulin V(H)II gene H8 /cds=(0,207) /gb=X02463 /gi=52418 /ug=Mm.88809 /len
3 539 Incl M35725:Mouse Cu-Zn superoxide dismutase mRNA, complete cds /cds=(4,468) /gb=M35725 /gi=192929 /ug=Mm.5274 /
4 1931 Incl L41495:Mus musculus protein-serine/threonine kinase (pim-2) mRNA, complete cds /cds=UNKNOWN /gb=L4149
5 6002 770.6 Cluster Incl AF016697:Duffy blood group /cds=(34,1038) /gb=AF016697 /gi=2454612 /ug=Mm.6393 /len=1174
6 6553 Incl AA200748:mu03a04.r1 Mus musculus cDNA, 5 end /clone=IMAGE-638286 /clone_end=5 /gb=AA200748 /gi=17
7 6953 Incl AI840815:UI-M-AH0-adb-c-03-0-UI.s1 Mus musculus cDNA, 3 end /clone=UI-M-AH0-adb-c-03-0-UI /clone_end=
8 8275 Incl AI849678:UI-M-AL1-ahl-e-08-0-UI.s1 Mus musculus cDNA, 3 end /clone=UI-M-AL1-ahl-e-08-0-UI /clone_end=3
9 8909 Incl AW061324:UI-M-BH1-anw-e-09-0-UI.s1 Mus musculus cDNA, 3 end /clone=UI-M-BH1-anw-e-09-0-UI /clone_en
10 9017 Incl AV280750:AV280750 Mus musculus cDNA, 3 end /clone=4933417D13 /clone_end=3 /gb=AV280750 /gi=626878
11 9322 4012.2 Cluster Incl AI851081:UI-M-BH0-akg-a-12-0-UI.s1 Mus musculus cDNA, 3 end /clone=UI-M-BH0-akg-a-12-0-UI /clone_end=
12 10673 Incl AI849834:UI-M-AL1-aho-h-09-0-UI.s1 Mus musculus cDNA, 3 end /clone=UI-M-AL1-aho-h-09-0-UI /clone_end=3
13 11080 Incl AI286904:ui78d09.y1 Mus musculus cDNA, 5 end /clone=IMAGE-1888529 /clone_end=5 /gb=AI286904 /gi=392
14 11516 Incl AI851048:UI-M-BH0-ajv-f-08-0-UI.s1 Mus musculus cDNA, 3 end /clone=UI-M-BH0-ajv-f-08-0-UI /clone_end=3 ,
15 12004 1749.2 Cluster Incl U43321 :Frizzled homolog 8, (Drosophila) /cds=(0,2057) /gb=U43321 /gi=1151259 /ug=Mm.57055 /len=2058
16 12047 3153.8 Cluster Incl X70764:ELKL motif kinase /cds=(93,2417) /gb=X70764 /gi=57919 /ug=Mm.4082 /len=2763
17 12300 834 Cluster Incl AV334165:AV334165 Mus musculus cDNA, 3 end /clone=6330554P20 /clone_end=3 /gb=AV334165 /gi=637421
